# Integration of tumor extrinsic and intrinsic features associates with immunotherapy response in non-small cell lung cancer

**DOI:** 10.1038/s41467-022-31769-4

**Published:** 2022-07-13

**Authors:** Denise Lau, Sonal Khare, Michelle M. Stein, Prerna Jain, Yinjie Gao, Aicha BenTaieb, Tim A. Rand, Ameen A. Salahudeen, Aly A. Khan

**Affiliations:** 1 Tempus Labs, Inc., Chicago, IL 60654 USA; 2 Department of Pathology, University of Chicago, Chicago, IL 60637 USA

**Keywords:** Tumour biomarkers, Non-small-cell lung cancer, Data integration, Tumour immunology, Translational research

## Abstract

The efficacy of immune checkpoint blockade (ICB) varies greatly among metastatic non-small cell lung cancer (NSCLC) patients. Loss of heterozygosity at the HLA-I locus (HLA-LOH) has been identified as an important immune escape mechanism. However, despite HLA-I disruptions in their tumor, many patients have durable ICB responses. Here we seek to identify HLA-I-independent features associated with ICB response in NSCLC. We use single-cell profiling to identify tumor-infiltrating, clonally expanded CD4^+^ T cells that express a canonical cytotoxic gene program and NSCLC cells with elevated HLA-II expression. We postulate cytotoxic CD4^+^ T cells mediate anti-tumor activity via HLA-II on tumor cells and augment HLA-I-dependent cytotoxic CD8^+^ T cell interactions to drive ICB response in NSCLC. We show that integrating tumor extrinsic cytotoxic gene expression with tumor mutational burden is associated with longer time to progression in a real-world cohort of 123 NSCLC patients treated with ICB regimens, including those with HLA-LOH.

## Abstract (web summary)

Some cancer patients with impaired HLA-I still respond to immunotherapy. Here the authors combine a cytotoxic gene signature from CD4+ and CD8+ T cells with tumor mutational burden to predict immunotherapy response in NSCLC patients, including those with HLA-LOH.

## Introduction

PD-1 and PD-L1 immune checkpoint blockade (ICB) has produced clinical responses in metastatic non-small cell lung cancer (NSCLC)^1,2^. However, clinical response rates and duration of response are highly variable, and the mechanisms of action of ICBs in patients remain poorly understood. Identifying patients who are likely to respond to a particular therapy is a cornerstone of personalized cancer medicine. Currently, tumor intrinsic features, such as tumor PD-L1 immunohistochemistry (IHC) and tumor mutational burden (TMB), are the most studied biomarkers for the prediction of response to ICB in NSCLC. While PD-L1 IHC staining has been clinically approved as a diagnostic for use in the front line to guide ICB therapy, it is complicated by the variety of assays and scoring criteria for measuring it. More notably, PD-L1 expression alone is insufficient to identify all potential responders to ICB in NSCLC^3,4^. Similarly, TMB lacks definitive clinical utility as large, randomized clinical trials have generated contradictory results^5–7^. Tumor extrinsic features such as the presence of tumor-infiltrating T cells have demonstrated some predictive value for ICB response^8,9^ but have yet to be independently assessed in NSCLC. Thus, there is an unmet clinical need for biomarkers that complement PD-L1 staining to better predict individualized outcomes for ICB in NSCLC.

With rapid advancements in clinical DNA and RNA sequencing and improved access to electronic health records, there is an increasing volume of real-world data that can be used to supplement clinical trial studies. These linked molecular and clinical real-world datasets can be used to evaluate transcriptional signatures or other genomic markers associated with cancer treatment outcomes in routine oncology practice. In this study, we evaluated the association between tumor intrinsic features and ICB outcomes by analyzing real-world molecular profiling data and outcomes from 123 NSCLC patients. In addition, we examined whether identifying and integrating tumor extrinsic features could more accurately predict the real-world clinical responses of NSCLC patients. A lack of NSCLC tumor biopsies with paired DNA and RNA sequencing data, as well as real-world outcome data, has hampered previous research into these questions.

An essential component in the model for ICB function is the direct killing of tumor cells by cytotoxic CD8^+^ T cells in response to tumor antigen presentation on HLA class I (HLA-I) molecules. Thus, disruption of HLA-I antigen presentation in tumors via somatic alterations, such as mutations in its co-receptor beta 2 microglobulin (*B2M*) or HLA-I loss of heterozygosity (HLA-LOH), has been proposed as an important mechanism of immune escape and resistance to ICB^10–12^. Accordingly, the rate of HLA-LOH has been reported to be as high as 40% in NSCLC patients^13^ and has been linked to worse survival on ICB^14,15^. However, evidence has emerged that patients with disrupted tumor HLA-I presentation can still have durable responses to ICB^9,16–19^. The mechanistic basis for ICB response in HLA-I disrupted tumors remains incompletely understood.

One potential mechanism for an HLA-I-independent immune response is through CD4^+^ T cells. Classically, effector CD4^+^ T cells help CD8^+^ T cells by licensing dendritic cells and secreting pro-inflammatory cytokines^20,21^. However, earlier studies in animal models have shown that CD4^+^ T cells, which recognize antigen via HLA class II (HLA-II) molecules rather than HLA-I, can also directly kill tumor cells^22,23^. Recently, studies using single-cell RNA sequencing (scRNAseq) have characterized antigen-specific cytotoxic CD4^+^ T cells in various cancers, including melanoma, breast, and colon cancer^24–26^. Furthermore, a population of cytotoxic CD4^+^ T cells was characterized in bladder cancer that directly killed autologous tumor cells in an HLA-II-dependent manner and was associated with improved response to ICB^27^. However, the presence, functions, and clinical implications of cytotoxic CD4^+^ T cells in NSCLC have not yet been characterized. Separately, multiple studies have found that HLA-II, which is typically expressed only on immune cells, can also be expressed on NSCLC tumor cells^28–30^, and tumor HLA-II expression has been linked to increased survival in NSCLC^31^. We postulate that cytotoxic CD4^+^ T cells exist in NSCLC and may function via HLA-II expression on tumor cells^32,33^, which, when viewed collectively, could be a mechanistic basis for anti-tumor immune responses observed in HLA-I-disrupted tumors.

In this work, we investigate the impact of HLA-LOH on ICB efficacy in a real-world patient cohort and identify HLA-I-independent features associated with ICB response in NSCLC. We use single-cell multi-omic profiling (a combination of scRNAseq, T cell receptor [TCR] sequencing, and surface protein profiling) to characterize the tumor and T cell compartments in NSCLC tumors from 10 patients. We identify a robust population of tumor-infiltrating, clonally expanded CD4^+^ T cells expressing a canonical cytotoxic gene program. Concordantly, we find tumor cells with elevated HLA-II expression in NSCLC patients. Following this discovery, we develop an integrative model by combining tumor extrinsic cytotoxic gene expression with TMB to predict ICB outcomes in a real-world cohort of 123 NSCLC patients, including those with HLA-I-disrupted tumors. Overall, this study recommends integrating tumor extrinsic and intrinsic features to more accurately model real-world clinical responses to immunotherapy in NSCLC.

## Results

### HLA class I-disrupted NSCLC patients can have durable responses to checkpoint inhibitors

To determine whether patients with HLA-I-disrupted NSCLC tumors can respond to ICB, we assembled a real-world cohort of 123 patients with metastatic, non-squamous NSCLC who were molecularly profiled prior to initiating standard ICB treatment. Patients with actionable *EGFR* or *ALK* alterations were excluded from the cohort. Patient tumor samples were profiled using targeted DNA sequencing^34,35^ or whole-exome sequencing, along with whole-transcriptome RNA sequencing^36,37^. Additionally, DNA sequencing was performed on matched normal samples obtained from blood or saliva when available (102/123). Response to therapy was evaluated using the time to progression (TTP)^38^ with a median cohort TTP of 210 days. In this cohort, 52% of patients received ICB as a monotherapy while the rest were treated in combination with chemotherapy, and 50% of patients were treated with ICB in the first line. Other key clinical metrics are detailed in Supplementary Data 1.

**Fig. 1:**
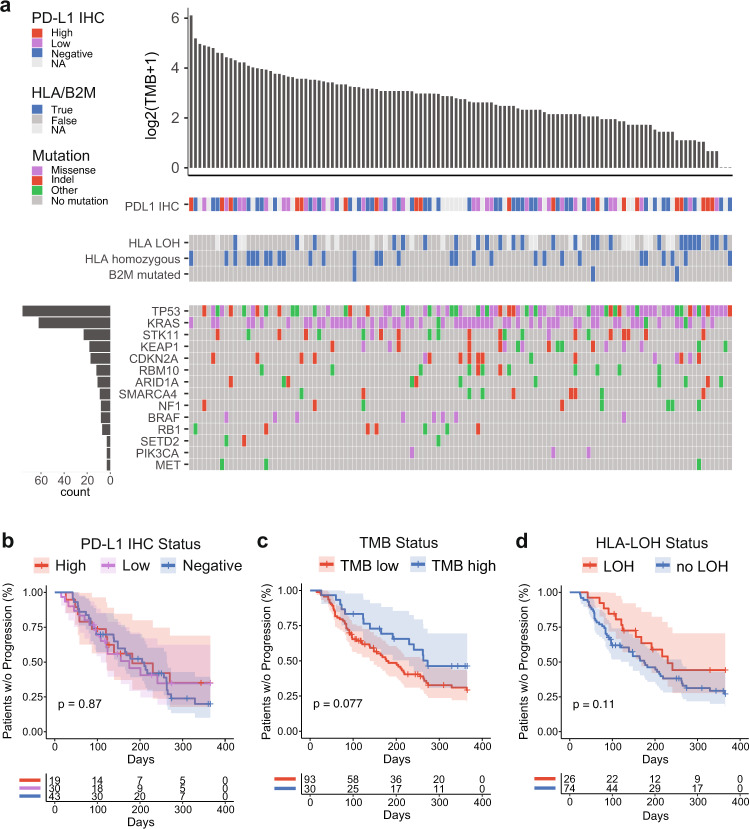
Cohort clinical and genomic characteristics. **a** Mutation plot showing the distribution of TMB and frequency of driver mutations across the cohort (*n* = 123). Each column represents a patient and columns are ordered by TMB. Immunotherapy biomarker status and mutation type are denoted by color. Genes are sorted by frequency of mutation. **b** Kaplan–Meier plots showing time to progression on ICB therapy, stratified by PD-L1 IHC status (*n* = 92, *p* = 0.87, log-rank), **c** TMB status (*n* = 123, HR = 0.60, *p* = 0.083, log-rank), and **d** HLA-LOH (*n* = 100, HR = 1.64, *p* = 0.11, log-rank). Source data are provided as a Source Data file.

The most frequently mutated driver genes were *TP53* (62%), followed by *KRAS* (50%) and *STK11* (19%) (Fig. 1a). Patients with *TP53*-mutated tumors had significantly longer TTP (HR = 0.60, *p* = 0.028, log-rank). We also assessed a number of previously described tumor intrinsic immunotherapy biomarkers, such as PD-L1 IHC, TMB, and HLA-LOH. Patients with PD-L1-high tumors (≥50% tumor cell staining) in this real-world cohort did not have improved TTP compared to PD-L1-negative and -low patients (HR = 0.86, *p* = 0.60, log-rank) (Fig. 1b). Tumors in this cohort had a median TMB of 5.71 mutations per megabase (mut/Mb). Patients with a high TMB (≥10 mut/Mb) had longer TTP than those with a low TMB (HR = 0.60, *p* = 0.077, log-rank) (Fig. 1c).

Similar to previous studies^13,14^, we found that a significant proportion of this real-world cohort had defects in class I antigen presentation, with 26% having LOH in at least one HLA-I gene and 2% harboring a *B2M* mutation (Fig. 1a, center). Furthermore, 24% of patients were homozygous for at least one HLA class I gene. Interestingly, patients in this cohort with HLA-LOH trended toward improved TTP compared to those with intact HLA-I (HR = 0.61, *p* = 0.11, log-rank). There was no significant association between *B2M* mutation or HLA homozygosity and TTP (*B2M*: HR = 1.24, *p* = 0.77, HLA homozygosity: HR = 0.88, *p* = 0.64) (Supplementary Fig. 1). These findings show that in a real-world setting, some patients with limitations in class I antigen presentation can still have durable clinical responses to immunotherapy.

### Identification of multiple cytotoxic T cell populations in NSCLC

**Fig. 2:**
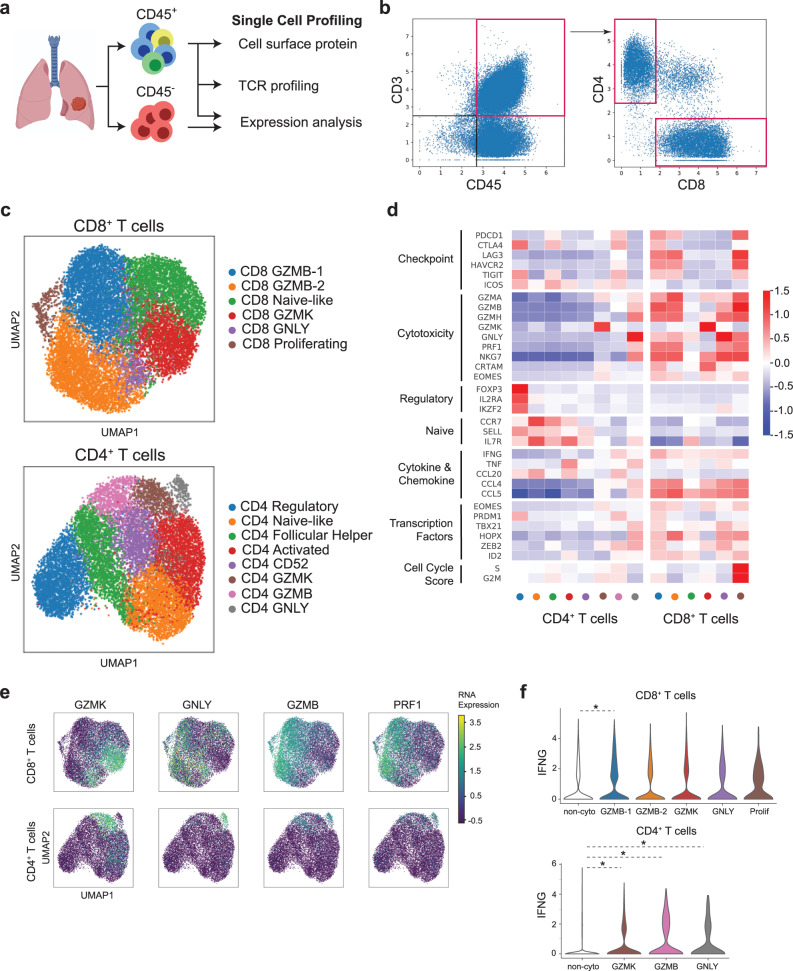
Single-cell characterization of tumor-infiltrating T cells. **a** Schematic overview of the experimental design (*n* = 10 tumor samples). **b** Gating strategy for the computational isolation of the CD4^+^ (12,711 cells) and CD8^+^ T cell compartment (13,935 cells). Expression is shown in centered log-ratio (CLR)-transformed counts. **c** UMAP projections show a subpopulation of cells in the T cell compartment based on Leiden clustering. Cells were labeled based on the expression of key immune function genes. **d** Heatmap showing the scaled expression of key immune function genes in the T cell compartment. **e** UMAP projections show the distinct patterns of expression for the cytotoxic genes *PRF1*, *GZMB*, *GZMK*, and *GNLY*. **f** Violin plots show the log-transformed RNA expression of *IFNG* in the Leiden clusters. *Represents clusters with significantly different expressions (*p* < 0.05) compared to the other clusters after Bonferroni correction (two-sided Wilcoxon Rank-Sum). For top panel: *p*_GZMB-1_ = 1.04 × 10^−23^, *n* = 13,935 cells; bottom panel: *p*_GZMK_ = 4.32 × 10^−12^, *p*_GZMB_ = 5.92 × 10^−44^, *p*_GNLY_ = 1.40 × 10^−5^, *n* = 12,711 cells). Source data are provided as a Source Data file.

To investigate the existence of HLA-I-independent mechanisms of ICB response, we sought to characterize the landscape of infiltrating T cells in the tumor-immune microenvironment of NSCLC. Single-cell profiling has become an important technique in investigating the heterogeneity of the human immune system and identifying clinically important cell types that would not be possible with bulk RNA expression profiling technology^39,40^. We performed single-cell profiling on 10 dissociated tumor samples obtained from patients with NSCLC who had never received treatment (Supplementary Data 2). Flow cytometry was used to separate samples into CD45^+^ and CD45^−^ fractions, which were then subjected to gene expression profiling via scRNAseq on the 10X Genomics Chromium platform. In addition, single-cell TCR and cell surface protein profiling using DNA-barcoded antibodies were performed on the CD45^+^  fraction (Fig. 2a). We profiled a total of 2806 CD45^−^ cells and 62,723 CD45^+^ cells. For our protein expression analysis, we computationally isolated the CD4^+^ T cell compartment (16,008 cells) and the CD8^+^ T cell compartment (13,935 cells) (Fig. 2b, Supplementary Fig. 2). After filtering on highly variable genes and normalizing the expression data to unit variance, we used Leiden clustering to identify subpopulations within the CD4^+^ and CD8^+^ T cell compartments that have distinct transcriptional programs (Fig. 2c).

Within the CD8^+^ T cell compartment, we identified six subpopulations. Notably, five of the six clusters, CD8_GZMK_, CD8_GNLY_, CD8_GZMB-1_, CD8_GZMB-2_, and CD8_prolif_ exhibited elevated expression of canonical cytotoxic genes, including granzymes and perforins, as well as associated transcription factors like *EOMES* (Fig. 2d). Cytotoxic T cells are critical effectors of immune response in solid tumors^41^. We found that the pattern of expression for specific cytotoxic genes varied across the CD8^+^ T cell compartment (Fig. 2e). *GZMA*, *GZMH*, and *NKG7* were expressed broadly across the cytotoxic CD8^+^ population, however, elevated expression of *GZMB* and *GZMK* was observed in distinct clusters (Fig. 2e, Supplementary Fig. 3). A prior study using peripheral blood showed that GZMK^+^ CD8^+^ T cells are at an earlier stage of maturation than GZMB^+^ CD8^+^ T cells^42^. Consistent with that finding, *CRTAM*, an immunoglobulin superfamily protein induced after TCR activation^43,44^, was primarily expressed in the CD8_GZMK_ population, indicating that these cells are in an earlier stage of effector cell differentiation than the other cytotoxic CD8^+^ populations. Interestingly, the CD8_GNLY_ cluster, which had the highest expression of the pore-forming peptide granulysin (*GNLY*), expressed both *GZMB* and *GZMK*, suggesting an intermediate state between the CD8_GZMK_ and CD8_GZMB_ clusters.

The two *GZMB*-high clusters identified, CD8_GZMB-1_ and CD8_GZMB-2_, shared a number of characteristics. They both exhibited elevated *CD103* and *CD39* expression, a phenotype that has previously been associated with an enrichment of tumor-reactive T cells^45,46^. In addition, they expressed high levels of immune checkpoint genes, such as *PDCD1*, *LAG3,* and *TIGIT*, suggesting that they are exhausted and could be targeted by ICB regimens. They also expressed *CXCL13*, which has been associated with better response and survival in NSCLC patients treated with PD-1 blockade due to enhanced immune cell recruitment to tertiary lymphoid structures^47^. Notably, the CD8_GZMB-1_ population was enriched for *FABP5* and *41BB* expressing cells, which is indicative of a heightened oxidative metabolic rate. A comparable population has been observed in hepatocellular carcinoma, and it is hypothesized that the metabolic adaptation of these cells confers a survival advantage in the tumor microenvironment^48^.

The final cytotoxic cluster, CD8_prolif_, appeared to be actively proliferating as evidenced by high S and G2M cell cycle scores relative to other clusters. These cells most likely represent clones that were recently TCR stimulated and are now undergoing active clonal expansion (Fig. 2d). The only non-cytotoxic cluster, CD8_naive-like_, expressed markers such as *CCR7* and *IL7R*, and most likely represent bystander T cells. All six populations, including the CD8_naïve-like_ cells, expressed *IFNG* (Fig. 2d, f). The IFN-γ-regulating transcription factors *EOMES* and *TBX21* were expressed in multiple populations, suggesting redundancy and overlapping transcriptional control of *IFNG*.

We next sought to characterize the relationship between the CD8^+^ clusters using partition-based graph abstraction (PAGA)^49^ (Supplementary Fig. 4a). Similar to our expectations based on the patterns of RNA expression, the CD8_GZMK_, and CD8_GNLY_ clusters had much greater connectivity to the CD8_naive-like_ than the two CD8_GZMB_ clusters. Interestingly, the CD8_prolif_ cluster was connected to the CD8_GZMB_ clusters but not the other cytotoxic clusters, suggesting that *GZMB* expressing cells are the primary CD8^+^ T cell population undergoing proliferation and clonal expansion in the tumor microenvironment.

We also identified populations of T cells with cytotoxic gene expression within the CD4^+^ T cell compartment, as previously noted in NSCLC^50^ and other cancers^24,27,51^. The pattern of cytotoxic gene expression was heterogeneous in these CD4^+^ T cells, but broadly paralleled that observed in the CD8^+^ T cell compartment (Fig. 2e). The expression of *GZMB* and *GZMK* was again elevated in distinct clusters (Fig. 2e, Supplementary Fig. 3). The *GNLY*-high cluster, CD4_GNLY_, co-expressed *GZMB* but not *GZMK*. Similarly, *CRTAM* and *EOMES*, which have been implicated in the induction of the cytotoxic program in CD4^+^ T cells^52,53^, were most highly expressed in the CD4_GZMK_ population. Interestingly, the CD4_GZMB_ cluster, but not the CD4_GZMK_ or CD4_GNLY_ clusters, expressed high levels of immune checkpoints such as *PDCD1* and *CTLA4* (Fig. 2d, Supplementary Fig. 3), and are therefore the cells likely to be responsive to ICB therapy^54^.

Notably, cytotoxic CD4^+^ T cells, particularly the CD4_GZMB_ and CD4_GNLY_ cells, expressed *IFNG* at significantly higher levels than non-cytotoxic CD4^+^ T cells (*p* < 0.0001 for both, Mann–Whitney *U*) (Fig. 2f). *IFNG* has been shown to directly increase HLA-II expression in tumor cells^23,30,31,55^. Thus, cytotoxic CD4^+^ T cells may serve as a reservoir for the paracrine induction of HLA-II antigen presentation machinery in tumor cells.

Consistent with other known functions of CD4^+^ T cells, we identified a T follicular helper cluster, CD4_follicular_, with high *IL7R* and *CD200* expression. Single-cell analyses in an NSCLC model have indicated that T follicular helper cells promote cytotoxic CD8^+^ T cell proliferation and tissue residence in the tumor microenvironment^56^. In addition, we also identified a regulatory T cell cluster, CD4_regulatory_, with high *FOXP3* expression and an activated T cell cluster, CD4_activated_, characterized by *CD69* expression (Fig. 2d).

We also sought to characterize the relationship between clusters in the CD4^+^ compartment using a PAGA graph (Supplementary Fig. 4b). Similar to the findings in CD8^+^ T cells, the CD4_GZMK_, and CD4_GNLY_ clusters had close connections to the CD4_naive-like_ cluster, but the CD4_GZMB_ cluster did not. Taken together, the comparison of CD8^+^ and CD4^+^ T cells reveals the distinct development of cytotoxic populations that share a number of transcriptional features, including increased levels of granzymes, perforin, and immune checkpoint genes.

### Cytotoxic T-cell populations are clonally expanded

Clonal expansion is a key trait of antigen-experienced T cells. To determine whether the cytotoxic T cell populations exhibited evidence of clonal expansion within the tumor microenvironment, we analyzed the TCR repertoires derived from the scRNAseq data. We define an expanded TCR clone as a population that contains more than one cell with identical TCR alpha and TCR beta CDR3 sequences and found evidence of extensive clonal expansion within all cytotoxic T cell populations.

**Fig. 3:**
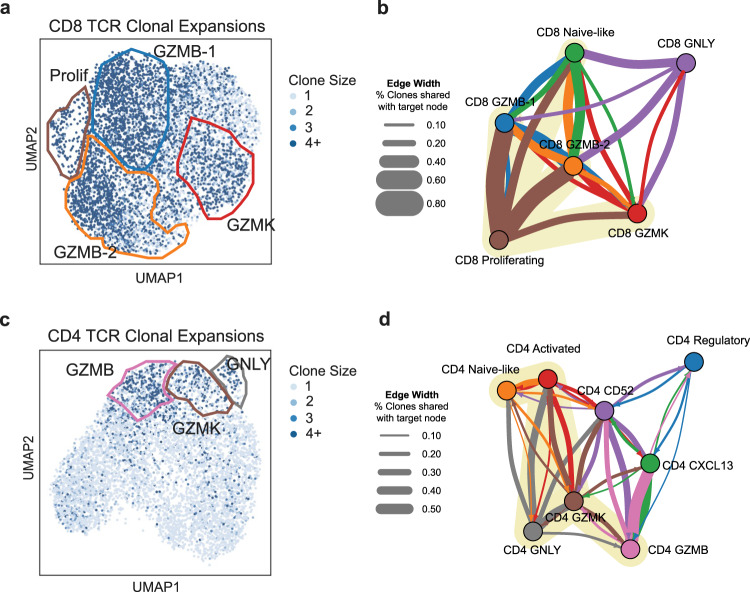
Cytotoxic CD4^+^ and CD8^+^ T cells are clonally expanded in NSCLC. **a** UMAP projection showing the clone size associated with the TCR for each cell in CD8^+^ and **c** CD4^+^ T cells. **b**, **d** TCR clonal association between phenotypic states visualized using a graph structure. Each node represents a Leiden cluster and the width of each directed edge represents the percent of clones from the starting node shared with the target node. The color of each edge matches that of the starting node. Edges with weights in the lowest tertile for CD4^+^ and lowest quartile for CD8^+^ T cells are not shown for clarity. Source data are provided as a Source Data file.

Almost all CD8^+^ cytotoxic cells (83%) were members of an expanded T cell clone, and the Shannon entropy of the TCR repertoire from cytotoxic CD8^+^ cells was significantly lower than that of non-cytotoxic CD8^+^ cells (*p* < 0.001, Hutcheson’s *t*-test) (Supplementary Fig. 5a). As expected, the CD8_prolif_ population was the most clonally expanded, followed by the CD8_GZMB-1_ and CD8_GZMB-2_ clusters (Fig. 3a, Supplementary Fig. 5b). Notably, the CD8_prolif_ cluster shared a high proportion of TCR clones with the two CD8_GZMB_ populations, indicating that these effector cells are being antigen-stimulated and actively undergoing clonal expansion (Fig. 3b). In contrast, the CD8_prolif_ cluster contained a lower proportion of CD8_GZMK_ and very few CD8_GNLY_ clones, implying that these early-stage cytotoxic CD8^+^ T cells proliferate less actively than CD8_GZMB_ populations.

There were far fewer clonally expanded CD4^+^ T cells than CD8^+^ T cells (Fig. 3c, Supplementary Fig. 5b). However, large clonal expansions were detected specifically within the cytotoxic CD4^+^ populations, with 54% of all cytotoxic CD4^+^ cells belonging to an expanded clone. Additionally, the Shannon entropy of the TCR repertoire of cytotoxic CD4^+^ cells was significantly lower than that of non-cytotoxic CD4^+^ cells (*p* < 0.001, Hutcheson’s *t*-test). This suggests that similar to cytotoxic CD8^+^ T cells, these cells are responding to tumor antigens.

When we looked at the prevalence of shared clones across CD4^+^ T cell subpopulations, we discovered that 26% of CD4_GZMK_ clones and 35% of CD4_GNLY_ clones were shared with CD4_activated_ cells (Fig. 3d). In contrast, only 3% of CD4_GZMB_ cell clones were shared with the CD4_activated_ population (Fig. 3d). This suggests CD4_GZMK_ and CD4_GNLY_ cells are likely earlier-stage cytotoxic cells that are differentiated from recently activated CD4^+^ cells. However, the CD4_GZMB_ population shared over 54% of its clones with the CD4_follicular_ population (Fig. 3d). T follicular helper cells have been hypothesized to promote anti-tumor CD8^+^ T cell responses in NSCLC^56^. The shared clonal lineage suggests that cytotoxic CD4^+^ T cells and those that may provision CD8^+^ T cells help stem from a common precursor. Taken together, our results demonstrate the significant clonal expansion of CD8^+^ and CD4^+^ cytotoxic T cells, suggesting that these populations both have encountered tumor antigens.

### NSCLC tumor cells express HLA class II

**Fig. 4:**
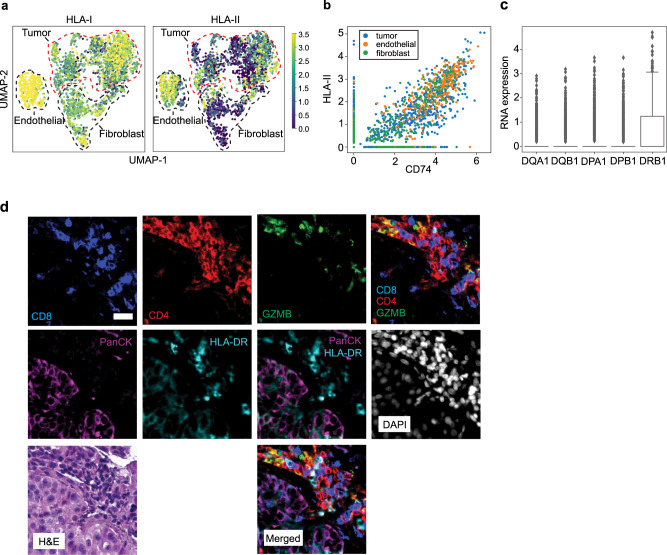
A subpopulation of tumor cells express HLA class II in NSCLC. UMAP projections showing the expression of **a** HLA-I and HLA-II in the CD45^−^ fraction. HLA-I and II expression was measured by summing the expression of all the individual genes assessed in the locus. **b** Comparison of HLA-II and *CD74* (invariant chain) expression (*R* = 0.751, unadjusted *p* = 0, Pearson correlation test). **c** Boxplots show the log-transformed expression of the individual HLA-II genes assessed (*p* = 1.01 × 10^−178^, Kruskal–Wallis, *n* = 1377 cells). The box represents the interquartile range, with the center line at the median. The whiskers extend up to 1.5 times the interquartile range (IQR). **d** Representative multiplex immunofluorescent staining of CD8 (blue), CD4 (red), GZMB (green), PanCK (magenta), HLA-DR (cyan), and DAPI (gray) in an NSCLC tumor (*n* = 2). Overlay without DAPI is shown for CD8, CD4, and GZMB, and for PanCK and HLA-DR, along with the corresponding H&E. Scale bar, 25 μm. Source data are provided as a Source Data file.

To determine whether NSCLC tumor cells have the ability to directly present antigen to cytotoxic CD4^+^ T cells, we evaluated HLA expression in the CD45^−^ cell fraction using scHLAcount^50^. We sequenced 2152 CD45^−^ cells and, utilizing Leiden clustering and lineage markers, identified distinct populations of the tumor, endothelial, and fibroblast cells (Supplementary Fig. 6). Since HLA-I is expressed by the vast majority of human cells, the majority of cells contained detectable amounts of HLA-I RNA (Fig. 4a). Expression of HLA-II is typically limited to antigen-presenting cells and endothelial cells. However, HLA-II has been shown to be expressed on lung epithelial cells^57,58^ as well as NSCLC tumor cells^28–30^. We found that a subset of tumor cells expressed HLA-II and that its expression was significantly correlated with that of its chaperone, *CD74* (invariant chain) (*R* = 0.627, *p* < 0.0001, Pearson correlation) (Fig. 4a, b). Notably, HLA-II expression in tumor cells was not uniform across the genes examined, with significantly higher *HLA-DRB1* than *HLA-DQA1*, *HLA-DQB1*, and *HLA-DPB1* (*p* < 0.0001, Kruskal–Wallis) (Fig. 4c).

We further investigated the presence of cytotoxic CD4^+^ T cells and HLA-II-expressing tumor cells in treatment-naive NSCLC samples using immunofluorescence (Fig. 4d, Supplementary Fig. 7). We tested and optimized a panel of tumor epithelial- and lymphocyte-specific markers. Pan-cytokeratin staining was used to identify epithelial populations within tumor samples, while CD4 and CD8 staining was used to identify tumor-infiltrating T cells. In addition, we used HLA-DR to evaluate HLA-II expression and GZMB to identify cytotoxic lymphocyte populations. We identified regions within tumor samples in which T cells co-expressed CD4 and GZMB, and tumor cells co-expressed pan-CK and HLA-DR. In some instances, close proximity was observed between these populations. Additionally, H&E staining of a neighboring slide revealed clusters of malignant cells adjacent to lymphocyte clusters. These findings indicate the possibility of direct physical interaction between cytotoxic CD4+ T cells and HLA-II-expressing NSCLC tumor cells.

### Integration of tumor extrinsic and intrinsic features associated with immunotherapy response

To determine the clinical relevance of CD8^+^ and CD4^+^ T cell cytotoxicity, we examined the relationship between the cytotoxic populations identified in the single-cell analysis and real-world ICB response in NSCLC. To begin, we generated gene signatures for each cluster by identifying the top 25 differentially expressed genes within cytotoxic clusters relative to all other clusters in the corresponding CD8^+^ and CD4^+^ compartments. We used the RNA-sequencing data from real-world patient tumor samples and their matched clinical data to assess the expression of specific gene signatures in patients and their association with ICB response. Cox proportional hazards (PH) analysis revealed that cytotoxic CD8^+^ and CD4^+^ signatures had the strongest associations with TTP (Supplementary Fig. 8a) (CD8_GZMB-1_: HR = 0.77, *p* = 0.033, CD4_GZMB_: HR = 0.80, *p* = 0.044, CoxPH). Thus, gene signatures from both cytotoxic CD8^+^ and CD4^+^ T cell populations were associated with real-world ICB response in NSCLC.

We next examined whether general CD8^+^ and CD4^+^ T cell infiltration were associated with real-world ICB response. We used estimates of CD8^+^ and CD4^+^ T cell infiltration derived from the xCell^59^ algorithm and found that TTP was not significantly associated with the presence of total CD8^+^ and CD4^+^ T cells (CD8^+^ T cells: HR = 0.86, *p* = 0.29, CD4^+^ T cells: HR = 1.11, *p* = 0.36, CoxPH) (Supplementary Fig. 8b). This demonstrated that the presence of cytotoxic T cells, rather than total T cells, is associated with better prognosis in patients with NSCLC treated with ICB.

Following these findings, we investigated whether a pan-T cell cytotoxicity signature could be used to predict response to ICB in NSCLC patients. We developed a canonical cytotoxic gene signature consisting of genes that were highly expressed by CD4^+^ or CD8^+^ cytotoxic T cells in our single-cell data (Supplementary Fig. 9a, b). The gene list includes 25 genes with the greatest log-fold change in expression in either CD4^+^ or CD8^+^ cytotoxic T cells compared to non-cytotoxic T cells. We calculated a cytotoxic score by taking the arithmetic mean of the selected genes’ log-transformed RNA expression values. Our cytotoxic score was found to be positively correlated with the gene signatures derived from the cytotoxic CD4^+^ and CD8^+^ Leiden clusters (Supplementary Fig. 9c). However, the cytotoxic score was not correlated to xCell estimates of total CD8^+^ or CD4^+^ T cell infiltration (Supplementary Fig. 9d, e).

**Fig. 5:**
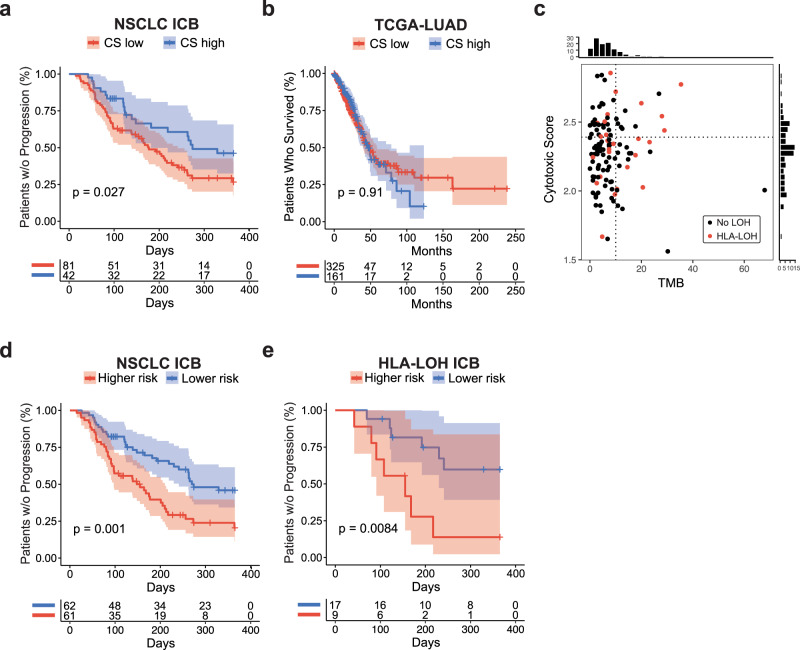
Cytotoxic gene signature is associated with ICB response in NSCLC. **a** Kaplan–Meier plots showing time to progression stratified by cytotoxic score (CS) status in the Tempus NSCLC ICB cohort (*n* = 123, HR = 0.56, *p* = 0.029, log rank), and **b** the TCGA-LUAD NSCLC cohort (*n* = 486, HR = 1.00, *p* = 1.0, log rank). **c** Correlation of TMB and CS in the NSCLC ICB cohort (*n* = 123, *R* = −0.0098, unadjusted *p* = 0.91, Pearson correlation test). **d** Kaplan–Meier plot showing time to progression on ICB therapy, stratified by multimodal score (MM) status in the Tempus NSCLC ICB cohort (*n* = 123, HR = 1.94, *p* = 0.005, log rank) and **e** the HLA-I-deficient subgroup (*n* = 26, HR = 3.59, *p* = 0.017, log rank). Source data are provided as a Source Data file.

Notably, the cytotoxic score was significantly associated with TTP in the Tempus ICB-treated NSCLC cohort (HR = 0.56, *p* = 0.027, log-rank) (Fig. 5a), but not in the TCGA LUAD^60^ cohort of patients primarily treated with platinum therapy (HR = 1.00, *p* = 1.0, log-rank) (Fig. 5b). This indicated that cytotoxic gene expression is an important tumor extrinsic characteristic associated with immunotherapy response.

We next considered various tumor intrinsic characteristics that may act as a complement to T cell cytotoxicity. We reasoned that a higher mutation burden would increase potential tumor antigen availability to cytotoxic CD4^+^ and CD8^+^ cells. Notably, the cytotoxic score was not correlated with TMB in the ICB-treated NSCLC cohort (*R* = −0.0028, *p* = 0.98, Pearson correlation) (Fig. 5c). We combined TMB and the cytotoxic score to create a simple complementary multimodal model (MM) capable of capturing both the intrinsic availability of potential tumor antigens and the infiltration of T cells with cytotoxic phenotypes. First, TMB was binarized using a 10 mut/Mb cutoff (Fig. 1c), a value previously used in clinical trials^5,8^. Then, we trained CoxPH models to predict TTP using the cytotoxic score and binarized TMB status on 100 random shuffles of the cohort, with 75% of patients in each shuffle used for training and 25% for evaluation. The model was evaluated using its out-of-fold performance. Each patient was assigned an MM score based on their average out-of-fold score, and the scores were binarized using the cohort’s median score as the cutoff to create two risk categories. In the Tempus ICB-treated NSCLC cohort, the MM score was significantly associated with TTP (HR = 2.17, *p* = 0.001, log-rank) (Fig. 5d).

Finally, we sought to specifically evaluate our MM performance on HLA-I-disrupted NSCLC patients. In the ICB-treated HLA-LOH subcohort, increasing MM score was significantly associated with shorter TTP (HR = 4.11, *p* = 0.0084, log-rank) (Fig. 5e). These findings demonstrate that features capturing T cell cytotoxic phenotype and TMB are associated with TTP in real-world ICB-treated NSCLC patients, including both HLA-I-intact and -disrupted patients.

## Discussion

ICB therapy is now used as a standard of care for the majority of metastatic NSCLC patients. In this study, we evaluated the association of tumor extrinsic features and intrinsic features with ICB response in a real-world cohort of NSCLC patients. We developed a gene signature for cytotoxicity that captures the transcriptional activity of both CD4^+^ and CD8^+^ cytotoxic T cells. Previous studies in other cancer types have demonstrated that cytotoxic gene signatures can be used as ICB biomarkers^41,61–63^, but the predictive ability of these signatures and their association with the CD4^+^ compartment have not been thoroughly explored in NSCLC. We demonstrate that patients with higher expression of our cytotoxic score had significantly longer TTP on ICB than those with lower expression. This metric was independent of TMB and had more predictive power than PD-L1 IHC status. These findings demonstrate that integrating a canonical cytotoxic gene signature with TMB may be an effective biomarker for identifying NSCLC patients who are more likely to respond to ICB and can remain robust even in populations with high levels of HLA-LOH or other HLA-I antigen presentation deficiencies.

In this study, we also characterized the tumor-infiltrating T cell compartment in 10 NSCLC tumors. We identified a CD4^+^ T cell population that expresses a canonical cytotoxic program similar to classical cytotoxic CD8^+^ T cells. Cytotoxic CD4^+^ T cells in cancer have primarily been characterized in animal models, though recent studies have identified them in patients across multiple cancer types^51^. However, the presence, functions, and clinical implications of these cells in NSCLC have remained largely unknown. In this work, we show that CD4^+^ T cells with a cytotoxic phenotype are a notable component of the tumor-infiltrating immune population in NSCLC. Additionally, we demonstrated these cells are clonally expanded, suggesting they are specific for a tumor antigen. These cells also express *IFNG*, which may induce HLA-II expression as observed in some NSCLC cells here. Given the evidence for cytotoxic CD4^+^ T cells in multiple solid tumors^51^ as well as now NSCLC, we propose that cytotoxic CD4^+^ T cells are a fundamental component of the tumor immune microenvironment. Recent work by Cohen et al.^54^ has elucidated the priming of CD4^+^ T cells that express cytotoxic genes (Tht-I) by antigen-presenting dendritic cells and implicated them in enhancing ICB-induced antitumor immune responses. At the same time, work by Oh et al. ^27^ have demonstrated cytotoxic CD4^+^ T cells can directly kill autologous tumors in an HLA-II-dependent manner. While anti-tumor activity in cancers lacking both HLA-II and HLA-I expression is thought to be coordinated by NK and CD4^+^ T cells^64,65^, we propose that the distinct expression of HLA-II on NSCLC cells presents a viable pathway for cytotoxic CD4^+^ T cells to drive ICB response in HLA-I-disrupted patients.

More research to further characterize cytotoxic CD4^+^ T cells will also facilitate the development of the next generation of ICB drugs. For example, therapeutically increasing HLA-II expression in myeloid or tumor cells may provide a novel way to rationally combine drugs with ICB treatment. The inhibition of CDK4/6 and MEK has been linked to increased HLA-II expression in tumor cells^66,67^. At the same time, cytotoxic CD4^+^ T cells upregulate *PDCD1*, *CTLA4*, and *TIGIT*, and are likely to be responsive to current ICB therapies. Notably, anti-CTLA-4 therapy, when combined with a decrease in regulatory T cells, has been shown to enhance cytotoxic CD4^+^ T cell anti-tumor activity^68^. Thus, such rational combinations may successfully enhance tumor antigen presentation and the efficacy of ICB in certain cancers.

We used real-world data to characterize the relationship between tumor extrinsic and intrinsic features and ICB response in NSCLC patients. While real-world data can more accurately reflect the complexities and diversity of patient care in the real world than clinical trials, it does have some drawbacks. Standard clinical trial endpoints, such as RECIST scores and overall survival, may be unavailable in some patients. Nonetheless, we were able to effectively investigate the impact of HLA-LOH on ICB efficacy and determine whether integrating tumor extrinsic and intrinsic features more accurately model real-world clinical responses^38^. Further clinical research will be required to validate our integrative model as a predictive or a broadly prognostic NSCLC biomarker for ICB. As a result, we believe that clinical trials and real-world data complement one another and that incorporating real-world data into clinical research can lead to more robust biomarker discovery and guide clinical trial designs.

Finally, the development of pre-treatment biomarkers continues to be an important goal for precision medicine. Such pre-treatment biomarkers can help inform clinical judgment and lead to better outcomes by identifying progression events sooner, limiting usage of ineffective and costly ICB regimens, and improving patient quality of life by potentially transitioning to the next line of therapy before asymptomatic progression becomes symptomatic progression. We note that biomarkers considering HLA-LOH and HLA diversity alone have produced contradictory results in predicting ICB outcomes^9,14,15,69^. Such HLA-I deficiencies are thought to limit tumor antigen presentation to CD8^+^ T cells but do not account for the potential role of cytotoxic CD4^+^ T cells. We propose that measuring the availability of potential tumor antigens and the infiltration of CD4^+^ and CD8^+^ T cells with cytotoxic phenotypes may aid in resolving the contradictory findings of these studies and provide a more complete picture of the tumor immune microenvironment. Overall, this study highlights the potential and utility of integrating tumor extrinsic and intrinsic features for predicting immunotherapy response in NSCLC.

## Methods

### Ethics statement

The use of de-identified molecular and clinical data in this study complies with all relevant ethical regulations. De-identified data were obtained from the Tempus Database. All data were de-identified in accordance with the Health Insurance Portability and Accountability Act (HIPAA) using Safe Harbor guidelines. The study protocol was submitted to the Advarra Institutional Review Board (IRB), which determined the research was exempt from IRB oversight.

### NSCLC ICB cohort and clinical endpoints

De-identified patient records were selected from the Tempus Database. For inclusion in this study, patients were required to (1) have a diagnosis of metastatic NSCLC with non-squamous histology, (2) have received an ICB regimen, (3) have a documented progression event after treatment initiation, or have at least 90 days of follow up from treatment initiation, (4) completed next-generation DNA and RNA sequencing on an ICB-naive biopsy, and (5) have no actionable *EGFR* or *ALK* alterations. The primary clinical endpoint was real-world time to progression (TTP), defined as the time from the initiation of the ICB regimen to the first progression event, censored on the last known clinical encounter^38^. Patients who ended treatment due to an adverse event, non-compliance, or another non-progression-related reason were censored at the time of treatment stop.

### Clinical data abstraction

Clinical features for this study were derived from unstructured physician progress notes. The physician notes were abstracted using a standardized enriched curation process. A data dictionary and template were developed and reviewed with a panel of oncologists. The data dictionary included every field, the associated value sets, the definition of the fields, and scenarios to clarify for abstraction. Each patient case was curated by two abstractors, blinded to each other’s curation. Discordances were reviewed by a third abstractor and escalated to a lung oncologist if adjudication was necessary. Two oncologists reviewed a random selection of patients from the final curated dataset to ensure validity.

### DNA and RNA sequencing

Formalin-fixed, paraffin-embedded (FFPE) patient samples were profiled using Tempus xT targeted panel (596 genes), Tempus xO targeted panel (1700 genes), or Tempus xE whole-exome DNA sequencing, as well as Tempus xT whole-transcriptome RNA sequencing. Each sample underwent expert pathologist assessment for tumor cellularity and other quality measures. All tumor samples had at least 20% tumor content. Total nucleic acid was extracted from the FFPE tissue sections and digested by proteinase K. RNA was purified from the total nucleic acid by DNase-I digestion. A matched normal sample of blood or saliva was also obtained when possible. Germline DNA was extracted from either 650 μl of saliva or 200 μl of blood. DNA and RNA sequencing were performed as previously described^34–36^.

### PD-L1 immunohistochemistry

PD-L1 status for the NSCLC ICB cohort was preferentially determined by clinical Tempus testing with the 22C3 anti-PD-L1 antibody (Agilent). Slides were scored by a pathologist using the tumor proportion score (TPS), which is the percentage of tumor cells with complete or partial membrane staining. Samples with a TPS <1% are considered PD-L1 negative, 1–49% are considered PD-L1 low, and ≥50% are considered PD-L1 high. For patients without Tempus 22C3 staining, PD-L1 IHC status was assessed from the abstracted clinical notes. For patients with recorded TPS scores, the same thresholds as described above were used. If no TPS score was available, but a PD-L1 IHC status was recorded, that status was used. PD-L1 IHC was performed on samples collected prior to ICB treatment start in all cases with known sample collection dates. Seven patients with abstracted PD-L1 IHC results had an unknown sample collection date. If there were multiple PD-L1 IHC tests recorded, the result from the test closest in time to the start of ICB treatment was used.

### TCGA LUAD cohort

FASTQ files from RNA sequencing data for the TCGA LUAD cohort were downloaded from the Genomic Data Commons^70^ and processed through the Tempus RNA pipeline as described below. The clinical data for the cohort was obtained from cBioportal^71^.

### Tumor mutational burden

TMB was calculated by dividing the number of non-synonymous mutations by the megabase size of the panel, as previously described^36^. All non-silent somatic coding mutations, including missense, indel, and stop-loss variants with coverage >100× and an allelic fraction >5% for targeted gene panels and coverage >30× and an allelic fraction >10% for the whole exome, were counted as non-synonymous mutations.

### HLA loss of heterozygosity

HLA-LOH status was determined as previously described^72,73^. Briefly, we assessed HLA-LOH status for all patients with a matched normal sample. Four-digit class, HLA-I typing was performed on the matched normal samples using Optitype (version 1.3.4)^74^ and a custom HLA reference file for each patient was generated. All reads mapped to the HLA locus, as well as unmapped reads were extracted from the tumor and normal BAM files and remapped to the patient’s HLA reference. After accounting for potential germline variants present in the sample’s HLA, the alignments were updated and allele-specific coverage was determined. Changes in coverage between alleles, in the context of the expected tumor purity and copy states in the flanking genome, were assessed to determine if any reduction in allele coverage was consistent with a clonal loss of a specific HLA allele.

### Transcriptomic analysis

Transcript-level quantification to GRCh37 was performed using Kallisto (version 0.44). Transcript counts were then corrected for GC content and length using quantile normalization and adjusted for sequencing depth via a size factor method. Normalized counts in protein-coding transcripts covered by the exome panel were then summed to obtain gene-level counts. Subsequent expression analyses were performed on log10-transformed counts.

### Single-cell multi-omics sequencing

Samples for single-cell multi-omic sequencing were previously frozen dissociated tumor cells (DTCs) (Discovery Life Sciences, Huntsville, AL). DTCs were thawed and washed with FACS buffer (PBS, 0.04% BSA). Up to 1 million cells were suspended in 45 μl cell staining buffer (BioLegend Cat. No. 420201). The cells were blocked with Human 5 μl TruStain FcX (Fc Receptor Blocking Solution, Cat. No. 422301) for 10 min on ice. An antibody pool was created by combining equal volumes of each Totalseq-C antibody, including the anti-Human-CD45 antibody (Cat# 368545, BioLegend), anti-Human-CD3 antibody (Cat# 300479, BioLegend), anti-Human-CD4 antibody (Cat# 300567, BioLegend), anti-Human-CD8 antibody (Cat# 344753, BioLegend), anti-Human-CD20 antibody (Cat# 302363, BioLegend), and FITC-conjugated anti-Human-CD45 antibody (Cat# 304006, BioLegend), into a pool. The final concentration of each antibody in the pool was 1 μg/μl as per the manufacturer’s recommendations. 1 μl (=1 μg) of mixed Totalseq-C + FITC-anti-CD45 antibody pool was then added to the 50 μl volume and the mixture was incubated for 30 min. on ice. Cells were washed by spinning at 400xG for 3 min, removing the upper liquid portion avoiding the pellet, and finally resuspension of the cell pellet in 500 μl cell staining buffer (first 2 washes). The third wash was with FACS buffer (PBS + 0.04% BSA) including DAPI (10 min RT incubation). Finally, the cells were resuspended in FACS buffer without DAPI pior to cell sorting.

Samples were sorted using the SH800S cell sorter (Sony Biotechnology). Live cells were gated on DAPI− cells sorted as CD45^+^ and CD45^−^ populations and collected in RPMI. The sorted CD45^+^ and CD45^−^ cells were pelleted and resuspended to recover a target of 3000 cells after 10X droplet formation. Cellular suspensions were barcoded using a Chromium Single Cell Controller instrument (10x Genomics) and 10X Genomics Chromium Single Cell A Chip Kit (P/N 120236, 10X Genomics) to generate single-cell Gel Beads-in-Emulsion (GEMs) for reverse transcription. Single-cell RNA-Seq libraries were prepared using the Chromium Single Cell 5’ Library and Gel Bead Kit (P/N 1000020, 10x Genomics) as per the manufacturer’s instructions. For each sample, four libraries were generated: CD45^−^ 5’ gene expression library, CD45^+^ 5’ gene expression library, CD45^+^ TCR library, and CD45^+^ cell surface protein library.

### Single-cell multi-omic analysis

Raw sequencing files were processed through the CellRanger pipeline (version 3.1.0) and then analyzed using Scanpy (version 1.6) and Scirpy (version 0.4). Cells with detectable gene expression in less than 200 genes, >6% mitochondrial genes for immune cells, >20% mitochondrial genes for tumor cells, or more than 2500 genes were removed from downstream analyses, as were any genes expressed in <3 cells. Scrublet^75^ was used for doublet detection and removal. Gene expression values were normalized to 10,000 counts per cell and log-transformed. Protein expression values were normalized using the centered log-ratio normalization.

Data from the CD45^+^ fraction was then filtered on the CD4^+^ and CD8^+^ T cell populations, based on protein expression of CD45, CD3, CD4, CD8, and CD20 and RNA expression of *CD68*. Data from the CD45^−^ fraction was filtered to remove a minor population of contaminating immune cells. Genes from the T cell receptor and HLA loci were removed from the gene expression data. The gene expression data was then batch corrected using BBKNN (version 1.5.1)^76^, filtered on highly variable genes, and scaled to unit variance. Leiden clustering was performed using Scanpy with a resolution of 0.7 for the CD8 cells and 0.8 for the CD4 cells. Cell cycle status was assessed using the score_genes_cell_cycle function in Scanpy with the gene list from Tirosh et al.^69^ PAGA graphs were generated with the paga function in Scanpy with 6 neighbors and the first 20 principal components. Differential expression analysis was performed using the rank_genes_groups function in Scanpy with the Wilcoxon Rank-Sum test.

To assess HLA expression, we first performed HLA typing using ArcasHLA (version 0.2.5)^77^ and then quantified HLA expression using scHLAcount (version 0.1.0)^78^. Gene-level raw counts were then normalized by library size and log-transformed.

For the T cell receptor analysis, cells without a paired TRA and TRB, or with multiple TRA or TRB chains were removed. A TCR clonotype was defined as a group of cells with identical TRA and TRB CDR3 sequences. An expanded clone was considered as any clone consisting of more than one cell. Shannon entropy was calculated using the alpha_diversity function in scirpy and Hutcheson’s *t*-test was performed using the ecolTest R package (version 0.0.1).

### Immunofluorescence staining

Multiplex immunofluorescence staining was performed on FFPE sections as previously described^79^. In brief, slides were deparaffinized and re-hydrated, followed by antigen retrieval. Slides were first stained with a cocktail of Tagged primary antibodies against CD8a (Clone: EPR10640(2), UltraTag: UT015, Cell Idx), CD4 (Clone: EPR6855, UltraTag: UT014, Cell Idx), Granzyme B (Clone: EPR20129-217, UltraTag: UT021, Cell Idx), panCK (Clone: AE1/AE3, UltraTag: UT016, Cell Idx), and HLA-DR (Clone: EPR3692, UltraTag: UT019, Cell Idx), and diluted with antibody diluent (PBS/1% BSA/0.2% Tween 20/15 mM Sodium Azide) for 1 h (UltraPlex detection system, Cell IDx). Slides were then washed with wash buffer and a cocktail of anti-Tag detection antibodies (UltraPlex detection system, Cell IDx), anti-UT015 (clone: CXC015, Flour: CL490, Cell Idx), anti-UT014 (clone: CXC015, Flour: CL550, Cell Idx), anti-UT021 (clone: CXC021, Flour: CL650, Cell Idx), anti-UT016 (clone: CXC016, Flour: CL480XL [megastoke dye], Cell Idx), and anti-UT019 (clone: CXC019, Flour: CL750, Cell Idx), were diluted with antibody diluent (PBS/1% BSA/0.2% Tween 20/15 mM Sodium Azide) and added to the slide and incubated for 1 h. As a negative control, slides were incubated with the secondary anti-Tag detection cocktail alone. Slides were then mounted using Fluoroshield with DAPI (Immunobiosciences) and coverslips applied prior to scanning at 20× using the Leica Versa scanner. Analysis was performed on the Aperio ImageScope, (v12.4.2.5010) using the Leica Quantitative Algorithm (v1).

### Gene signatures

Gene lists for the Leiden cluster gene signatures were generated by taking the top 25 ranked differentially expressed genes for each cluster using the rank_genes_group Scanpy function. The gene score was then calculated by taking the arithmetic mean of the log-transformed RNA expression of the gene list. Scores were mean-centered and scaled prior to use in survival analyses.

The cytotoxic score was calculated by taking the arithmetic mean of 25 genes that are highly expressed in either CD4^+^ or CD8^+^ cytotoxic T cells. The genes are *NKG7, CXCL13, GZMH, HAVCR2, CCL5, GZMK, CCL4, GZMA, CCL3, CST7, CCL4L2, ACP5, TNFRSF9, TIGIT, GZMB, PDCD1, PRF1, LYST, SIRPG, LAG3, CARD16, TUBA4A, PTMS, CD74, KLRD1*.

### Immune infiltration estimates

Estimates of immune cell infiltration were generated using the R package xCell^59^ (version 1.1.0).

### Multimodal model training

The MM score was generated via a Cox proportional hazards (CoxPH) model using cytotoxic score and the binarized TMB status to predict TTP on 100 random shuffles of the cohort. The train_test_split function from sklearn was used to randomly split 75% of patients in each shuffle into the training set and 25% of patients into the evaluation set. The CoxPHSurvivalAnalysis function from the scikit-survival (version 0.17.2)^80^ package was used to train the CoxPH models with the alpha parameter set to 1 and Breslow tie handling. Each patient was scored based on the arithmetic mean of their out-of-fold scores. The scores were then binarized using the median score of the cohort as the threshold to create two risk categories.

### Survival analysis

Kaplan–Meier plots were generated using the survminer R package (version 0.4.8). Forest plots were generated using the survivalAnalysis R package (version 0.1.3). The log-rank test was used to compare survival curves and hazard ratios were calculated using a Cox proportional hazards model.

### Reporting summary

Further information on research design is available in the Nature Research Reporting Summary linked to this article.

## Supplementary information

Supplementary Information

Description of Additional Supplementary Files

Supplementary Data 1

Supplementary Data 2

Reporting Summary

## Data Availability

Patient demographics data are available in Supplementary Data 1 and 2. The raw sequencing data are not publicly available due to data privacy regulations and commercial restrictions on the use of such data. As such, the genomic data analyzed here, including the de-identified clinical data, DNA variant data, RNA expression data and single-cell multiomic profiling data, is available and may be obtained in accordance with Tempus’s data sharing policy (https://vivli.org/ourmember/tempus/) as part of an external data access request (Accession ID: T21.02) linked here: 10.25934/PR00007504. The approximate time for processing a data access request is one month and inquiries about the process can be directed to publications@tempus.com. The TCGA data used in this study are available on the NIH Genomic Data Commons (https://portal.gdc.cancer.gov/projects/TCGA-LUAD) and the cBioPortal (https://www.cbioportal.org/study/summary?id=luad_tcga_pan_can_atlas_2018). The remaining data are available within the Article, Source Data, and Supplementary Information and Data files. Source data are provided with this paper.

## Source data

Source Data

## Code availability

Requests for code used to generate the figures from Source Data are subject to a non-commercial data software agreement and review by Tempus and can be directed to publications@tempus.com. All other code used in this study is previously published and specified in the “Methods” section.
