# Supplementary material for: Integration of tumor extrinsic and intrinsic features associates with immunotherapy response in non-small cell lung cancer
Source: Nat Commun. 2022 Jul 13;13:4053. doi: 10.1038/s41467-022-31769-4 (PMC9279502; doi:10.1038/s41467-022-31769-4)
Supplement: Supplementary file 1 — Supplementary Information [file 41467_2022_31769_MOESM1_ESM.pdf]

Supplementary Information for

Integration of tumor extrinsic and intrinsic features associates with immunotherapy response in non-small cell lung cancer

Denise Lau, Sonal Khare, Michelle M. Stein, Prerna Jain, Yinjie Gao, Aicha Ben-Taib, Tim A. Rand, Ameen Salahudeen, Aly A. Khan

Includes:

Supplementary Fig. 1-9

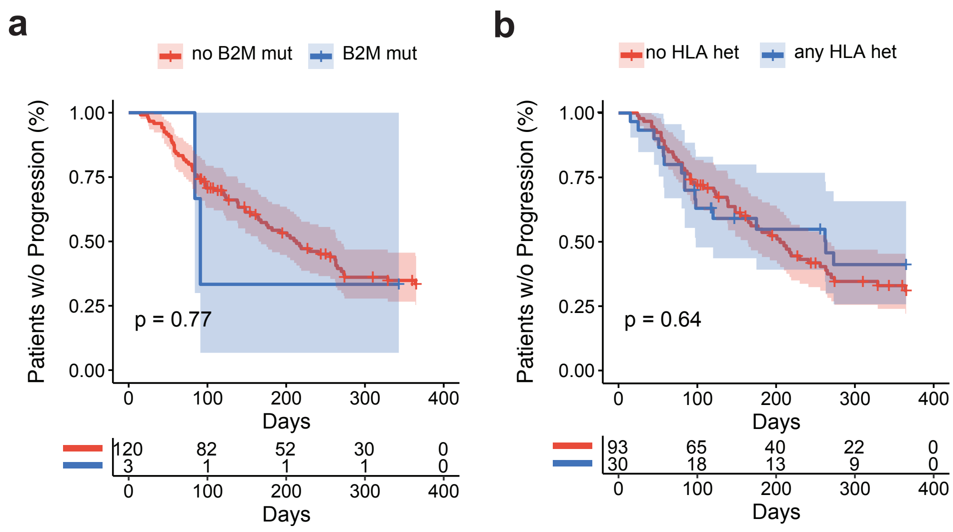
a
no B2M mut
B2M mut
Patients w/o Progression (%)
1.00
0.75
0.50
0.25
0.00
p = 0.77
0
100
200
300
400
Days
120
82
52
30
0
3
1
1
1
0
0
100
200
300
400
Days
b
no HLA het
any HLA het
Patients w/o Progression (%)
1.00
0.75
0.50
0.25
0.00
p = 0.64
0
100
200
300
400
Days
93
65
40
22
0
30
18
13
9
0
0
100
200
300
400
Days

Supplementary Fig. 1: **HLA class I deficiencies and TTP in the NSCLC ICB cohort. a,** Kaplan-Meier plots showing time to progression on ICB therapy, stratified by *B2M* mutation (n=123, HR=1.24, p=0.77, log rank), and **b,** HLA homozygosity status (n=123, HR=0.88, p=0.64, log rank). Source data are provided as a Source Data file.

a

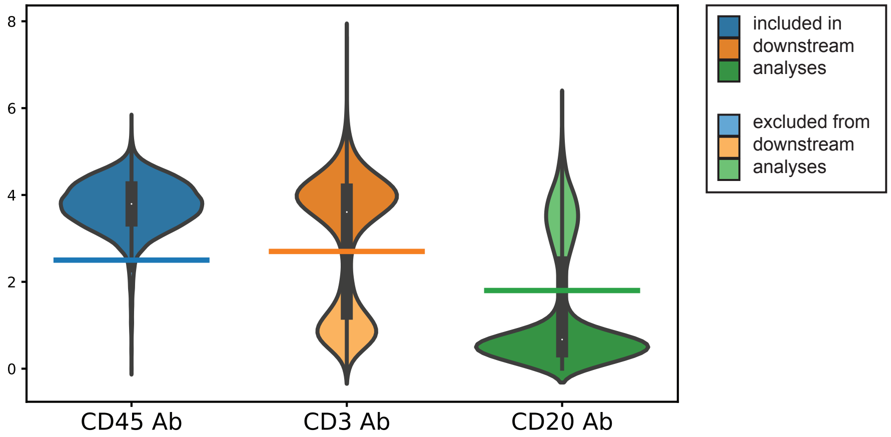

8
6
4
2
0
CD45 Ab
CD3 Ab
CD20 Ab
included in
downstream
analyses
excluded from
downstream
analyses

b

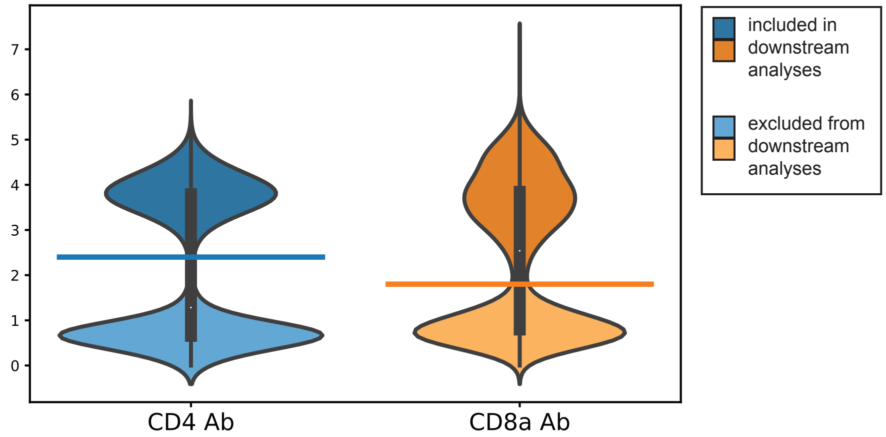

7
6
5
4
3
2
1
0
CD4 Ab
CD8a Ab
included in
downstream
analyses
excluded from
downstream
analyses

Supplementary Fig. 2: **Protein expression distribution of lineage markers. a**, Violin plots of normalized protein expression distribution of CD45 (blue), CD3 (orange) and CD20 (green) in 47,991 cells. Box inside each violin represents the interquartile range (IQR), whiskers extend up to 1.5 times the interquartile range (IQR). The point in white represents median expression. Each line indicates the threshold used to computationally isolate T cells for downstream analysis, where area of violin in bold color was included, and lighter region was excluded. **b**, Violin plots of normalized protein expression distribution (n=29,391 cells) of CD4 (blue) and CD8a (orange) following isolation of T cells using the thresholds in panel a. Each line indicates the threshold used to computationally isolate cells. Area of violin in bold color was considered CD4+ (using CD4) or CD8+ (CD8a). Box inside each violin represents the interquartile range (IQR), whiskers extend up to 1.5 times the interquartile range (IQR). The point in white represents median expression. Source data are provided as a Source Data file.

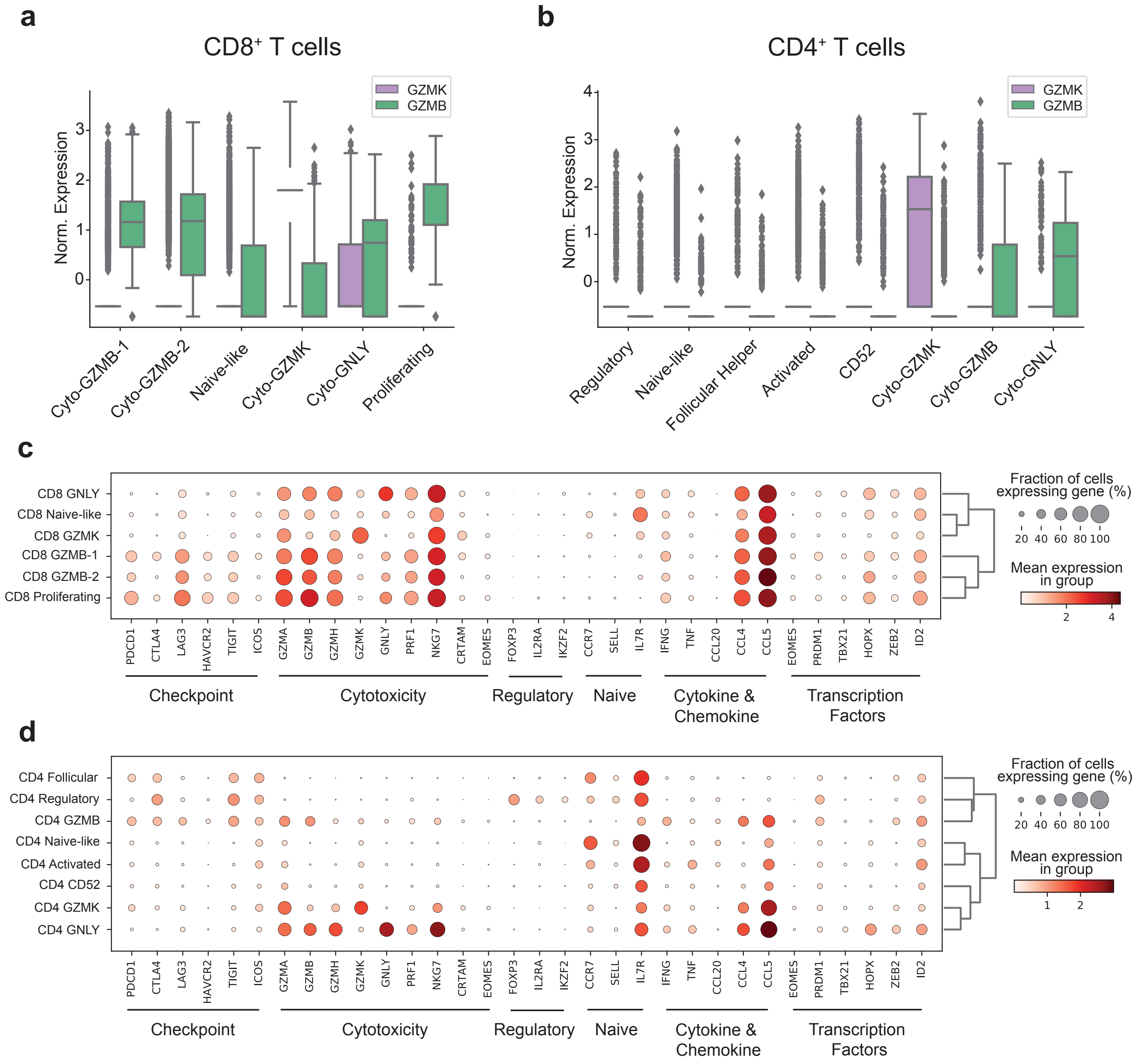

a
CD8⁺ T cells
GZMK
GZMB
Norm. Expression
Cyto-GZMB-1
Cyto-GZMB-2
Naive-like
Cyto-GZMK
Cyto-GNLY
Proliferating
b
CD4⁺ T cells
GZMK
GZMB
Norm. Expression
Regulatory
Naive-like
Follicular Helper
Activated
CD52
Cyto-GZMK
Cyto-GZMB
Cyto-GNLY
c
CD8 GNLY
CD8 Naive-like
CD8 GZMK
CD8 GZMB-1
CD8 GZMB-2
CD8 Proliferating
PDCD1 CTLA4 LAG3 HAVCR2 TIGIT ICOS GZMA GZMB GZMH GZMK GNLY PRF1 NKG7 CRTAM EOMES FOXP3 IL2RA IKZF2 CCR7 SELL IL7R IFNG TNF CCL20 CCL4 CCL5 EOMES PRDM1 TBX21 HOPX ZEB2 ID2
Checkpoint
Cytotoxicity
Regulatory
Naive
Cytokine & Chemokine
Transcription Factors
Fraction of cells expressing gene (%)
20 40 60 80 100
Mean expression in group
2 4
d
CD4 Follicular
CD4 Regulatory
CD4 GZMB
CD4 Naive-like
CD4 Activated
CD4 CD52
CD4 GZMK
CD4 GNLY
PDCD1 CTLA4 LAG3 HAVCR2 TIGIT ICOS GZMA GZMB GZMH GZMK GNLY PRF1 NKG7 CRTAM EOMES FOXP3 IL2RA IKZF2 CCR7 SELL IL7R IFNG TNF CCL20 CCL4 CCL5 EOMES PRDM1 TBX21 HOPX ZEB2 ID2
Checkpoint
Cytotoxicity
Regulatory
Naive
Cytokine & Chemokine
Transcription Factors
Fraction of cells expressing gene (%)
20 40 60 80 100
Mean expression in group
1 2

Supplementary Fig. 3**: *GZMB* and *GZMK* expression in CD4$^+$ and CD8$^+$ T cells. a,** Normalized RNA expression of *GZMK* and *GZMB* for each Leiden cluster in the CD8+ (Cyto-GZMB-1: 3933 cells; Cyto-GZMB-2: 3743 cells; Naive-like: 2779 cells; Cyto-GZMK: 2350 cells; Cyto-GNLY: 811 cells; Proliferating: 319 cells) and **b**, CD4$^+$ T cell populations (Regulatory: 2524 cells; Naive-like: 2398 cells; Follicular Helper: 2138 cells; Activated: 2085 cells; CD52: 1426 cells; Cyto-GZMK: 944 cells; Cyto-GZMB: 911 cells; Cyto-GLNY: 285 cells). The box in the boxplot represents the interquartile range, with the center line at the median. The whiskers extend up to 1.5 times the interquartile range (IQR). **c**, Bubble plot showing the expression of key immune function genes, including cytotoxic genes, in the CD8$^+$ T cell and **d**, the CD4$^+$ T cell Leiden clusters. Bubble size reflects the fraction of cells that have non-zero expression of the gene and the color represents the mean expression of the population. Source data are provided as a Source Data file.

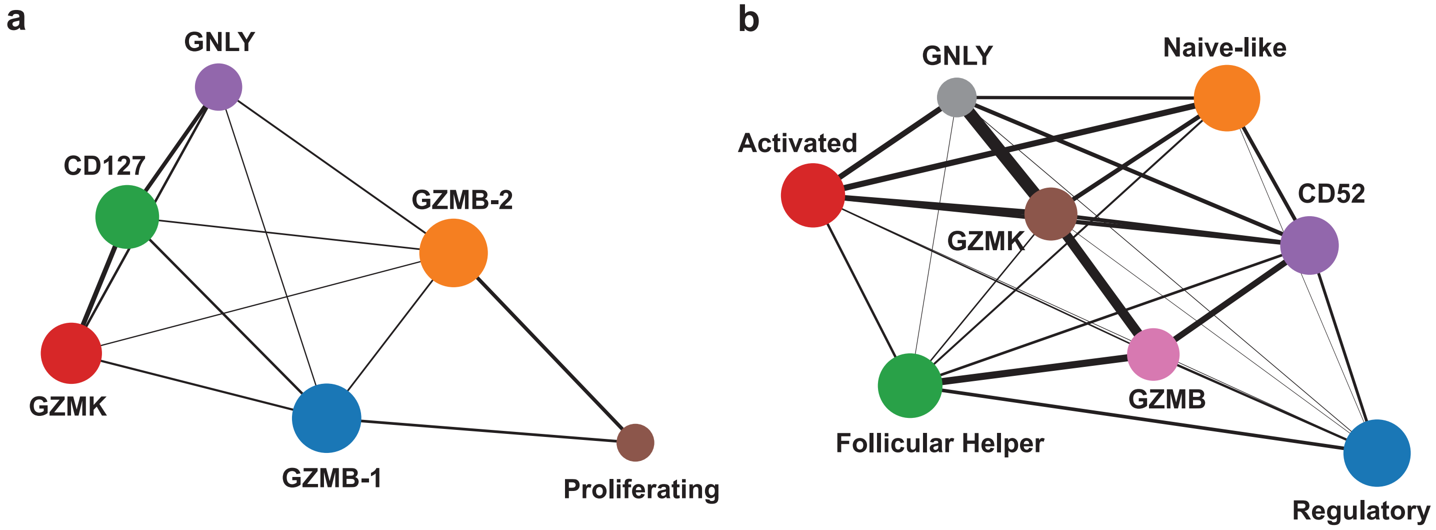

a
GNLY
CD127
GZMB-2
GZMK
GZMB-1
Proliferating
b
GNLY
Naive-like
Activated
CD52
GZMK
GZMB
Follicular Helper
Regulatory

Supplementary Fig. 4: **Trajectory analysis of $CD4^+$ and $CD8^+$ T cell populations. a**, PAGA graph of the $CD8^+$ and **b**, $CD4^+$ T cell populations. The nodes represent the Leiden clusters and the edges represent a statistical measure of connectivity based on RNA expression. The node size reflects the number of cells belonging to the cluster.

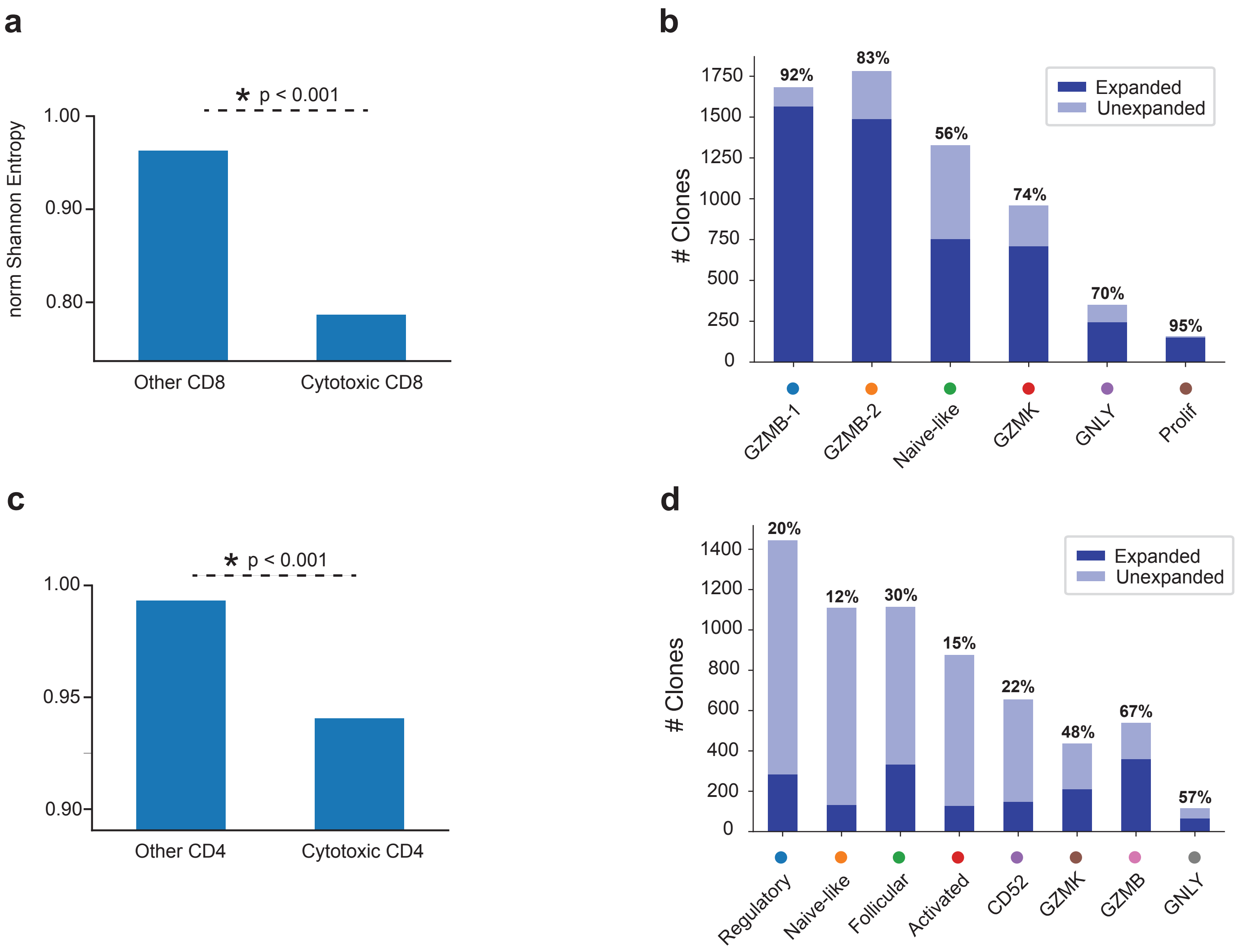

a
* p < 0.001
norm Shannon Entropy
1.00
0.90
0.80
Other CD8
Cytotoxic CD8
b
Clones
1750
1500
1250
1000
750
500
250
0
92%
83%
56%
74%
70%
95%
Expanded
Unexpanded
GZMB-1
GZMB-2
Naive-like
GZMK
GNLY
Prolif
c
* p < 0.001
1.00
0.95
0.90
Other CD4
Cytotoxic CD4
d
Clones
1400
1200
1000
800
600
400
200
0
20%
12%
30%
15%
22%
48%
67%
57%
Expanded
Unexpanded
Regulatory
Naive-like
Follicular
Activated
CD52
GZMK
GZMB
GNLY

Supplementary Fig. 5: **Clonal expansion in CD4$^+$ and CD8$^+$ T cells. a**, Normalized Shannon entropy for the CD8$^+$ cells and **c**, CD4$^+$ cells in cytotoxic clusters compared to those in the non-cytotoxic clusters (CD8$^+$: p=0, CD4+: p=0, two-sided Hutcheson's t-test). Proportion of clones that were expanded (more than one cell) for each Leiden cluster in **b,** CD8$^+$ and **d,** CD4$^+$ T cells. The percentage of expanded clones is shown above each bar. Source data are provided as a Source Data file.

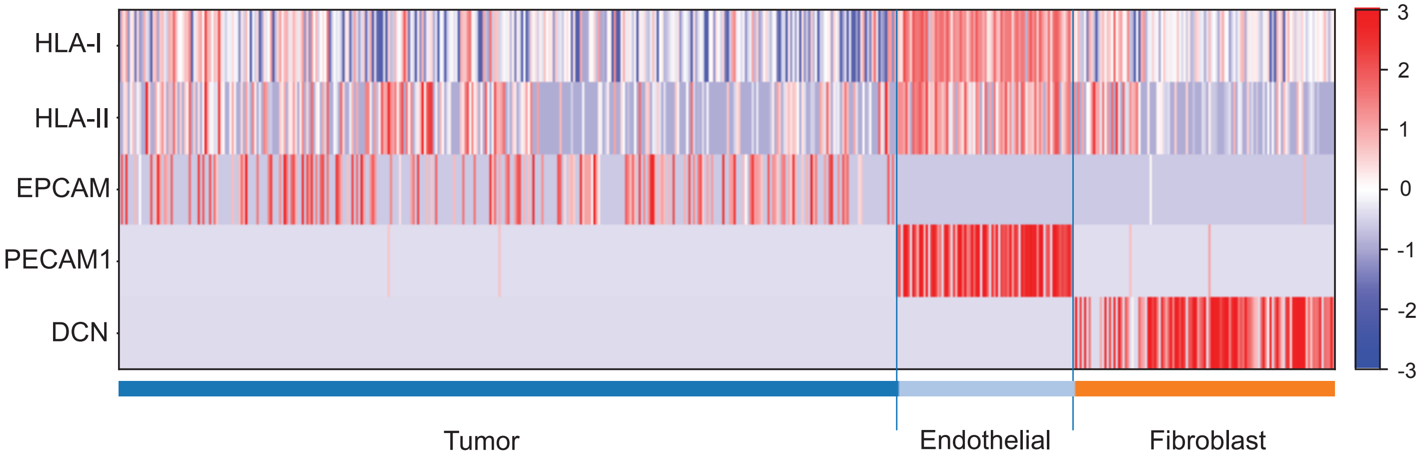

HLA-I
HLA-II
EPCAM
PECAM1
DCN
3
2
1
0
-1
-2
-3
Tumor
Endothelial
Fibroblast

Supplementary Fig. 6: **HLA and lineage marker expression in the CD45$^-$ population.** Heatmap showing the expression of HLA genes, as well as the lineage markers used to identify the tumor, endothelial and fibroblast populations in the CD45$^-$ fraction. Each column represents one cell. RNA expression is mean centered and scaled. Source data are provided as a Source Data file.

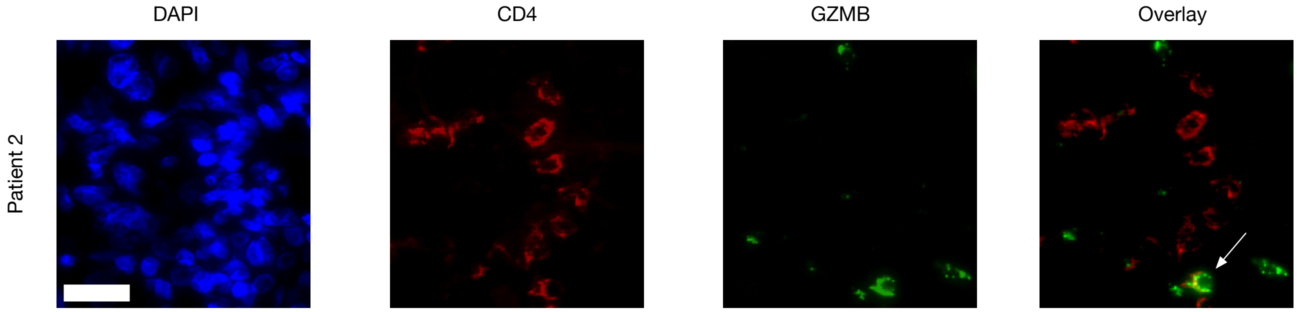
DAPI
CD4
GZMB
Overlay
Patient 2

Supplementary Fig. 7. **Additional example of GZMB protein expression in $CD4^+$ T cells.**

Representative multiplex immunofluorescent staining of DAPI (blue), CD4 (red), GZMB (green) in an NSCLC tumor (n = 2). Scale bar, 25 μm.

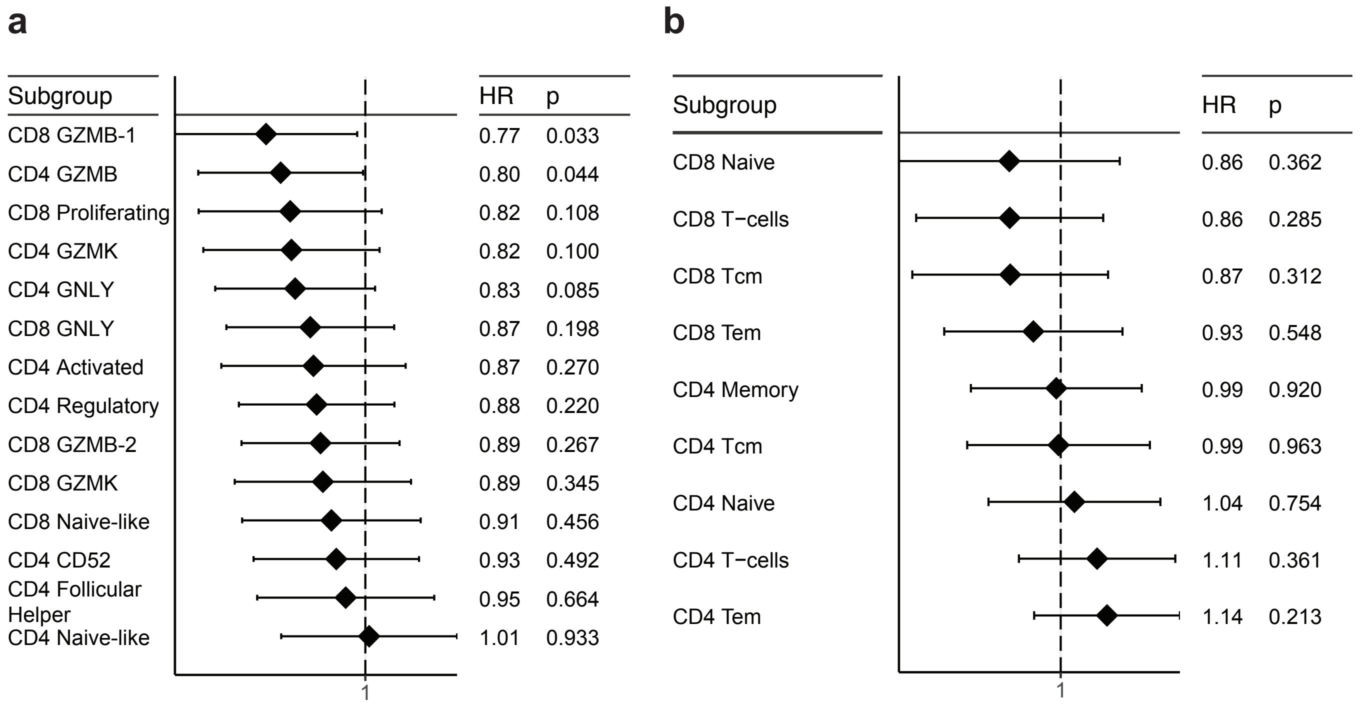
a
Subgroup
HR
p
CD8 GZMB-1 0.77 0.033
CD4 GZMB 0.80 0.044
CD8 Proliferating 0.82 0.108
CD4 GZMK 0.82 0.100
CD4 GNLY 0.83 0.085
CD8 GNLY 0.87 0.198
CD4 Activated 0.87 0.270
CD4 Regulatory 0.88 0.220
CD8 GZMB-2 0.89 0.267
CD8 GZMK 0.89 0.345
CD8 Naive-like 0.91 0.456
CD4 CD52 0.93 0.492
CD4 Follicular Helper 0.95 0.664
CD4 Naive-like 1.01 0.933
1
b
Subgroup
HR
p
CD8 Naive 0.86 0.362
CD8 T−cells 0.86 0.285
CD8 Tcm 0.87 0.312
CD8 Tem 0.93 0.548
CD4 Memory 0.99 0.920
CD4 Tcm 0.99 0.963
CD4 Naive 1.04 0.754
CD4 T−cells 1.11 0.361
CD4 Tem 1.14 0.213
1

Supplementary Fig. 8: **Association between T cell sub-populations and ICB outcomes.** Forest plots showing the univariate Cox proportional hazards models of TTP and expression of **a**, gene signatures for Leiden clusters, and **b**, xCell estimates of T cell infiltration in the Tempus NSCLC ICB cohort (n=123). Forest plots reflect results for mean-centered and scaled gene signatures and infiltration estimates and are not adjusted for multiple testing. The diamond represents the hazard ratio (HR) while the bars show the 95% confidence intervals. Source data are provided as a Source Data file.

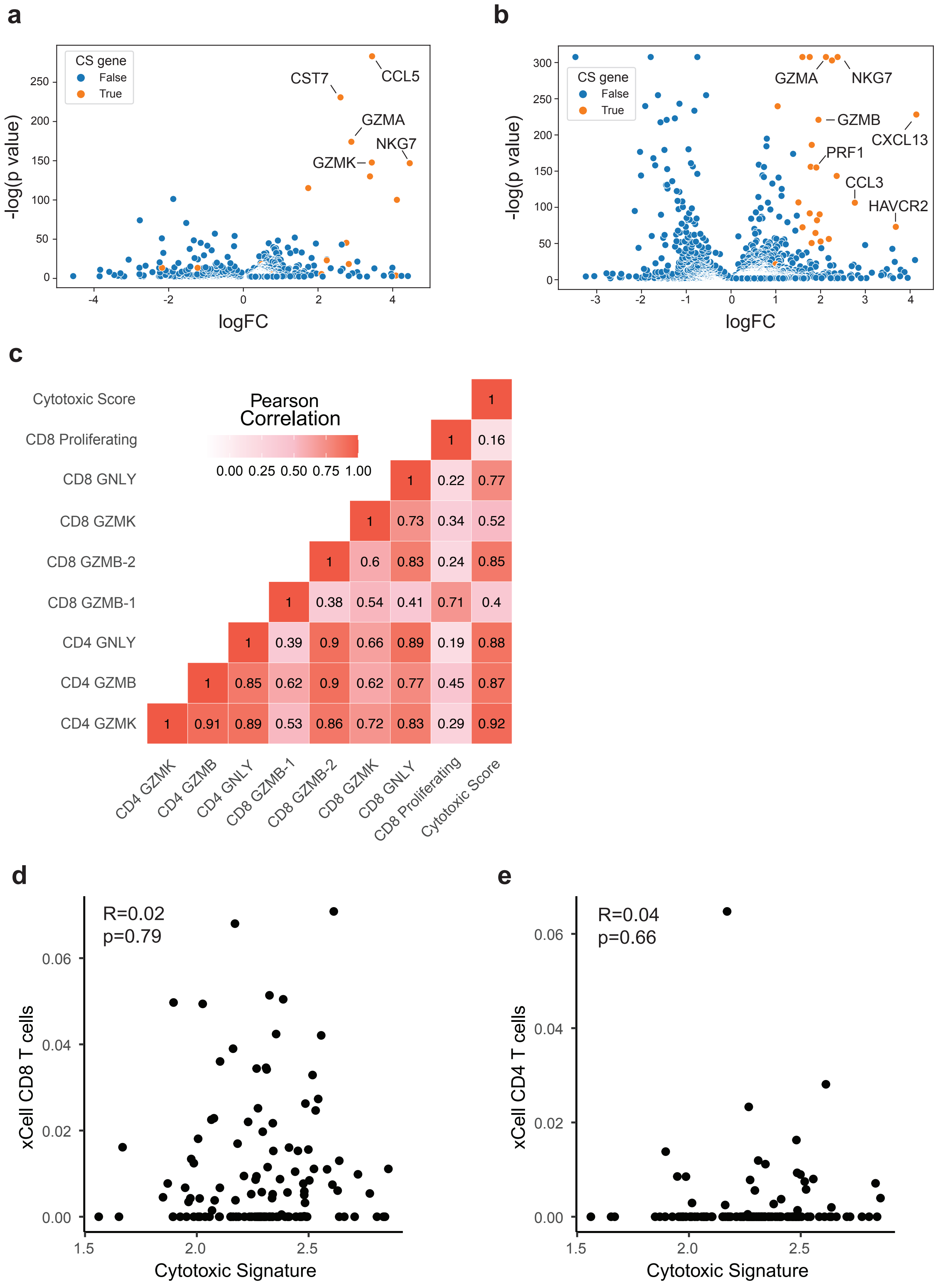

	CD4 GZMK	CD4 GZMB	CD4 GNLY	CD8 GZMB-1	CD8 GZMB-2	CD8 GZMK	CD8 GNLY	CD8 Proliferating	Cytotoxic Score
Cytotoxic Score									1
CD8 Proliferating								1	0.16
CD8 GNLY							1	0.22	0.77
CD8 GZMK						1	0.73	0.34	0.52
CD8 GZMB-2					1	0.6	0.83	0.24	0.85
CD8 GZMB-1				1	0.38	0.54	0.41	0.71	0.4
CD4 GNLY			1	0.39	0.9	0.66	0.89	0.19	0.88
CD4 GZMB		1	0.85	0.62	0.9	0.62	0.77	0.45	0.87
CD4 GZMK	1	0.91	0.89	0.53	0.86	0.72	0.83	0.29	0.92

Supplementary Fig. 9: **Cytotoxic score (CS) is positively correlated with the presence of $CD4^+$ and $CD8^+$ cytotoxic T cell populations.** Volcano plot showing the log fold change and Benjamini-Hochberg adjusted -log10 p-values of genes in cytotoxic T cell clusters compared to non-cytotoxic T cell clusters in the **a**, $CD8^+$ and **b**, $CD4^+$ T cell compartments. Expression differences were assessed by Wilcoxon rank sum test. Genes in the CS gene list are colored orange and key genes are labelled. **c**, Heatmap showing the correlation between CS and the gene signatures for cytotoxic T cell populations identified from Leiden clustering in the Tempus NSCLC ICB cohort (n=123). Each box is labelled with the Pearson correlation coefficient. **d**, **e** Scatter plots showing the correlation between xCell estimates of $CD8^+$ T cell and $CD4^+$ T cell infiltration (CD8: R=0.02, p=0.79, CD4: R=0.04, p=0.66, Pearson correlation test, no adjustments for multiple comparisons) in the NSCLC ICB cohort (n=123). Source data are provided as a Source Data file.
