# Supplementary material for: Integration of tumor extrinsic and intrinsic features associates with immunotherapy response in non-small cell lung cancer
Source: Nat Commun. 2022 Jul 13;13:4053. doi: 10.1038/s41467-022-31769-4 (PMC9279502; doi:10.1038/s41467-022-31769-4)
Supplement: Supplementary file 2 — Description of Additional Supplementary Files [file 41467_2022_31769_MOESM2_ESM.pdf]

Supplementary Data 1: Tempus NSCLC ICB cohort characteristics 5

Supplementary Data 2: Single-cell sample characteristics
