# Supplementary material for: Integration of tumor extrinsic and intrinsic features associates with immunotherapy response in non-small cell lung cancer
Source: Nat Commun. 2022 Jul 13;13:4053. doi: 10.1038/s41467-022-31769-4 (PMC9279502; doi:10.1038/s41467-022-31769-4)
Supplement: Supplementary file 5 — Reporting Summary [file 41467_2022_31769_MOESM5_ESM.pdf]

nature portfolio

Corresponding author(s): Denise Lau, Aly A. Khan

Last updated by author(s): Jun 8, 2022

Reporting Summary

Nature Portfolio wishes to improve the reproducibility of the work that we publish. This form provides structure for consistency and transparency in reporting. For further information on Nature Portfolio policies, see our Editorial Policies and the Editorial Policy Checklist.

Statistics

For all statistical analyses, confirm that the following items are present in the figure legend, table legend, main text, or Methods section.

n/a	Confirmed	
☐	☒	The exact sample size (n) for each experimental group/condition, given as a discrete number and unit of measurement
☐	☒	A statement on whether measurements were taken from distinct samples or whether the same sample was measured repeatedly
☐	☒	The statistical test(s) used AND whether they are one- or two-sided<br>*Only common tests should be described solely by name; describe more complex techniques in the Methods section.*
☐	☒	A description of all covariates tested
☐	☒	A description of any assumptions or corrections, such as tests of normality and adjustment for multiple comparisons
☐	☒	A full description of the statistical parameters including central tendency (e.g. means) or other basic estimates (e.g. regression coefficient) AND variation (e.g. standard deviation) or associated estimates of uncertainty (e.g. confidence intervals)
☐	☒	For null hypothesis testing, the test statistic (e.g. F, t, r) with confidence intervals, effect sizes, degrees of freedom and P value noted<br>*Give P values as exact values whenever suitable.*
☒	☐	For Bayesian analysis, information on the choice of priors and Markov chain Monte Carlo settings
☒	☐	For hierarchical and complex designs, identification of the appropriate level for tests and full reporting of outcomes
☐	☒	Estimates of effect sizes (e.g. Cohen's d, Pearson's r), indicating how they were calculated

Our web collection on statistics for biologists contains articles on many of the points above.

Software and code

Policy information about availability of computer code

Data collection	De-identified clinical and molecular data was obtained through the Tempus Labs Real-World Evidence database (Methods). Flow collection was performed using SH800S cell sorter (Sony Biotechnology). Cellular suspensions were barcoded using a Chromium Single Cell Controller instrument (10x Genomics). All sequencing was performed on the Illumina HiSeq 4000 (Illumina).
Data analysis	Python (version 3.9.7.) and R (version 4.2) code is available in packages as described in the manuscript (Methods). Software used: Kallisto (version 0.44) (https://pachterlab.github.io/kallisto/); Optitype (version 1.3.4) (https://github.com/FRED-2/OptiType); CellRanger (version 3.1.0) (https://support.10xgenomics.com/single-cell-gene-expression/software/downloads/latest); Scanpy (version 1.6) (https://github.com/theislab/scanpy); BBKNN (version 1.5.1) (https://github.com/Teichlab/bbknn); Scirpy (version 0.4) (https://icbi-lab.github.io/scirpy/latest/); ArcasHLA (version 0.2.5) (https://github.com/RabadanLab/arcasHLA); scHLAcount (version 0.1.0) (https://github.com/10XGenomics/scHLAcount); survminer (version 0.4.8) (https://cran.r-project.org/web/packages/survminer/index.html); scikit-survival (0.17.2) (https://scikit-survival.readthedocs.io/en/stable/); HLA LOH algorithm is described in Larson et al, Cell Reports 2021; Variant calling pipeline is described in Beaubier et al, Nat. Biotech 2019 and Beaubier et al, Oncotarget 2019

For manuscripts utilizing custom algorithms or software that are central to the research but not yet described in published literature, software must be made available to editors and reviewers. We strongly encourage code deposition in a community repository (e.g. GitHub). See the Nature Portfolio guidelines for submitting code & software for further information.

Data

Policy information about availability of data

All manuscripts must include a data availability statement. This statement should provide the following information, where applicable:

- Accession codes, unique identifiers, or web links for publicly available datasets
- A description of any restrictions on data availability
- For clinical datasets or third party data, please ensure that the statement adheres to our policy

Patient demographics data are available in Supplementary Data 1 and 2. The genomic data analyzed here, including the de-identified clinical data, DNA variant data, RNA expression data and single-cell multiomic profiling data, is available and may be obtained in accordance with Tempus's IP data sharing policy as part of an external data access request: https://vivli.org/ourmember/tempus/; Accession ID: T21.02. The TCGA data used in this study are available on the NIH Genomic Data Commons (https://gdc.cancer.gov) and the cBioPortal (https://www.cbioportal.org). The remaining data are available within the Article, Source Data, and Supplementary Information and Data files.

Field-specific reporting

Please select the one below that is the best fit for your research. If you are not sure, read the appropriate sections before making your selection.

☒ Life sciences ☐ Behavioural & social sciences ☐ Ecological, evolutionary & environmental sciences

For a reference copy of the document with all sections, see nature.com/documents/nr-reporting-summary-flat.pdf

Life sciences study design

All studies must disclose on these points even when the disclosure is negative.

Sample size	Sample size was not determined before the study. This was a retrospective study, and all qualified patients prior to September 2020 were selected from the de-identified Tempus Database.
Data exclusions	For inclusion in this study, patients were required to 1) have a diagnosis of metastatic NSCLC with a non-squamous histology, 2) have received an ICB regimen, 3) have a documented progression event after treatment initiation or have at least 90 days of follow up from treatment initiation, 4) completed next-generation DNA and RNA sequencing on a ICB-naïve biopsy, and 5) have no actionable EGFR or ALK alterations. The primary clinical endpoint was real-world time to progression (TTP), defined as the time from the initiation of the ICB regimen to the first progression event, censored on the last known clinical encounter. Patients who ended treatment due to an adverse event, non-compliance, or another non-progression related reason were censored at the time of treatment stop.
Replication	This was a retrospective study, and all qualified patients prior to September 2020 were selected from the de-identified Tempus Database. Thus, replication is not applicable as these are individual retrospective patient samples.
Randomization	This was a retrospective study, and all qualified patients prior to September 2020 were selected from the de-identified Tempus Database. Thus, randomization of patients was not applicable.
Blinding	This was a retrospective study, and all qualified patients prior to September 2020 were selected from the de-identified Tempus Database. Thus, blinding of patients was not applicable.

Reporting for specific materials, systems and methods

We require information from authors about some types of materials, experimental systems and methods used in many studies. Here, indicate whether each material, system or method listed is relevant to your study. If you are not sure if a list item applies to your research, read the appropriate section before selecting a response.

Materials & experimental systems

n/a	Involved in the study	
☐	☒	Antibodies
☒	☐	Eukaryotic cell lines
☒	☐	Palaeontology and archaeology
☒	☐	Animals and other organisms
☐	☒	Human research participants
☒	☐	Clinical data
☒	☐	Dual use research of concern

Methods

n/a	Involved in the study	
☒	☐	ChIP-seq
☒	☐	Flow cytometry
☒	☐	MRI-based neuroimaging

Antibodies

Antibodies used	Antibodies for cell sorting:<br>FITC-conjugated anti-Human-CD45 antibody (Cat# 304006, BioLegend)<br><br>Antibodies for single cell protein profiling<br>Totalseq-C anti-Human-CD45 antibody (Cat# 368545, BioLegend)<br>Totalseq-C anti-Human-CD3 antibody (Cat# 300479, BioLegend)<br>Totalseq-C anti-Human-CD4 antibody (Cat# 300567, BioLegend)<br>Totalseq-C anti-Human-CD8 antibody (Cat# 344753, BioLegend)<br>Totalseq-C anti-Human-CD20 antibody (Cat# 302363, BioLegend)<br><br>An antibody pool was created by combining equal volumes of each Totalseq-C antibody, including anti-Human-CD45 antibody (Cat# 368545, BioLegend), anti-Human-CD3 antibody (Cat# 300479, BioLegend), anti-Human-CD4 antibody (Cat# 300567, BioLegend), anti-Human-CD8 antibody (Cat# 344753, BioLegend), anti-Human-CD20 antibody (Cat# 302363, BioLegend), and FITC-conjugated anti-Human-CD45 antibody (Cat# 304006, BioLegend), into a pool. The final total antibody pool concentration was 1 µg/µl . 1µl (=1 µg) of mixed Totalseq-C + FITC-anti-CD45 antibody pool was then added to the 50 µl volume and the mixture was incubated for 30 min. on ice.<br><br>Primary antibodies for multiplex IF<br>Cell IDx anti-Human CD8a antibody (Clone: EPR10640(2), UltraTag: UT015)<br>Cell IDx anti-Human CD4 antibody (Clone: EPR6855, UltraTag: UT014)<br>Cell IDx anti-Human Granzyme B antibody (Clone: EPR20129-217, UltraTag: UT021)<br>Cell IDx anti-Human panCK antibody (Clone: AE1 / AE3, UltraTag: UT016)<br>Cell IDx anti-Human HLA-DR antibody (Clone: EPR3692, UltraTag: UT019)<br><br>Secondary antibodies for multiplex IF<br>Cell IDx anti-UT015 antibody (clone: CXC015, Flour: CL490)<br>Cell IDx anti-UT014 antibody (clone: CXC015, Flour: CL550)<br>Cell IDx anti-UT021 antibody (clone: CXC015, Flour: CL650)<br>Cell IDx anti-UT016 antibody (clone: CXC015, Flour: CL480XL [megastoke dye])<br>Cell IDx anti-UT019 antibody (clone: CXC015, Flour: CL750)<br><br>Slides were first stained with a cocktail of Tagged primary antibodies against CD8a (Clone: EPR10640(2), UltraTag: UT015, Cell Idx), CD4 (Clone: EPR6855, UltraTag: UT014, Cell Idx), Granzyme B (Clone: EPR20129-217, UltraTag: UT021, Cell Idx), panCK (Clone: AE1 / AE3, UltraTag: UT016, Cell Idx), and HLA-DR (Clone: EPR3692, UltraTag: UT019, Cell Idx), and diluted with antibody diluent (PBS/1% BSA/0.2% Tween 20/15 mM Sodium Azide) for 1 h (UltraPlex detection system, Cell IDx). Slides were then washed with wash buffer and a cocktail of anti-Tag detection antibodies (UltraPlex detection system, Cell IDx), anti-UT015 (clone: CXC015, Flour: CL490, Cell Idx), anti-UT014 (clone: CXC015, Flour: CL550, Cell Idx), anti-UT021 (clone: CXC021, Flour: CL650, Cell Idx), anti-UT016 (clone: CXC016, Flour: CL480XL [megastoke dye], Cell Idx), and anti-UT019 (clone: CXC019, Flour: CL750, Cell Idx), were diluted with antibody diluent (PBS/1% BSA/0.2% Tween 20/15 mM Sodium Azide) and added to the slide and incubated for 1 hour.
Validation	Representative figures are present in Figure 2. Additional validation is present on the manufacturers website, including https://www.biolegend.com/en-us/quality/quality-control, and https://cellidx.com/products/ultratag/multiplex-labeling-kit.

Human research participants

Policy information about studies involving human research participants

Population characteristics	This was a retrospective study, and all qualified patients prior to September 2020 were selected from the de-identified Tempus Database. Please see Supplementary Table 1 for additional details.
Recruitment	No patients were recruited specifically for this study. De-identified data was obtained from the Tempus Database.
Ethics oversight	The use of de-identified molecular and clinical data in this study complies with all relevant ethical regulations. All data were de-identified in accordance with the Health Insurance Portability and Accountability Act (HIPAA) using Safe Harbor guidelines. The study protocol was submitted to the Advarra Institutional Review Board (IRB), which determined the research was exempt from IRB oversight.

Note that full information on the approval of the study protocol must also be provided in the manuscript.
